# Supplementary material for: Discovery and substrate specificity engineering of nucleotide halogenases
Source: Nat Commun. 2024 Jun 19;15:5254. doi: 10.1038/s41467-024-49147-7 (PMC11186838; doi:10.1038/s41467-024-49147-7)
Supplement: Supplementary file 1 — Supplementary Information [file 41467_2024_49147_MOESM1_ESM.pdf]

Supplementary Information for

Discovery and Substrate Specificity Engineering of Nucleotide Halogenases

Jie Ni[1], Jingyuan Zhuang[1], Yiming Shi[1], Ying-Chih Chiang[2], and Gui-Juan Cheng[1]*

[1]Warshel Institute for Computational Biology, School of Medicine, The Chinese University of Hong Kong, Shenzhen, 518172, Guangdong, China.

[2] Kobilka Institute of Innovative Drug Discovery, School of Medicine, The Chinese University of Hong Kong, Shenzhen, 518172, Guangdong, China.

*Corresponding to chengguijuan@cuhk.edu.cn

This PDF file includes:

1. Supplementary Figures 6

1.1 Identification of new dGMP nucleotide halogenases – Figure S1 to S18 6

1.2 Binding mode and mechanism of nucleotide specificity – Figure S19 to S32 18

1.3 Engineering of nucleotide specificity– Figure S33 to S40 26

1.4 Role of the second-sphere residues – Figure S41 to S51 33

2. Supplementary Tables 41

3. Supplementary Notes 44

3.1 Primers used in this study 44

3.2 DNA and amino acid sequences 45

3.3 ^{1}H NMR and ^{13}C NMR of 2′-Cl-dGMP 46

4. Supplementary References 46

List of supplementary figures:

Supplementary Figure 1. Sequence alignment of VaNTH and CtNTH with AdaV analogs. 7
Supplementary Figure 2. SDS-PAGE analysis of VaNTH and CtNTH. 7
Supplementary Figure 3. Substrate panel of natural nucleotides and nucleoside triphosphate. 8
Supplementary Figure 4. HPLC analysis of enzymatic reactions catalyzed by CtNTH with substrates 4-8. 8
Supplementary Figure 5. LC-HRMS analysis of the chlorination products 2′-Cl-dGMP. 9
Supplementary Figure 6. LC-HRMS analysis of the chlorination products 2′-Cl-dIMP. 9
Supplementary Figure 7. LC-HRMS analysis of the chlorination products 2′-Cl-dAMP. 10
Supplementary Figure 8. ^{1}H NMR spectrum of **1a** (2′-Cl-dGMP) (500MHz, DMSO-*d6*). 10
Supplementary Figure 9. ^{13}C NMR spectrum of **1a** (2′-Cl-dGMP) (125MHz, DMSO-*d6*). 11
Supplementary Figure 10. HSQC spectrum of **1a** (2′-Cl-dGMP). 11
Supplementary Figure 11. NOESY spectrum of **1a** (2′-Cl-dGMP). 12
Supplementary Figure 12. Steady-state kinetic analysis for VaNTH, CtNTH, and AdaV with dGMP and dAMP substrates. 13
Supplementary Figure 13. HPLC analysis of enzymatic reactions catalyzed by CtNTH with dGMP and NaBr/NaI/NaN_3/$NaNO_2$. 14
Supplementary Figure 14. LC-HRMS analysis of enzymatic reactions catalyzed by CtNTH with dGMP and NaBr/NaN_3/$NaNO_2$ 15
Supplementary Figure 15. HPLC analysis of enzymatic reactions catalyzed by CtNTH with dGMP and NaOCN/NaSCN. 15
Supplementary Figure 16. LC-HRMS analysis of the bromination products 2′-Br-dGMP. 16
Supplementary Figure 17. LC-HRMS analysis of the azidation products 2′-N_3-dGMP. 16
Supplementary Figure 18. LC-HRMS analysis of the hydroxylation product GMP. ..17
Supplementary Figure 19. Relative activities of CtNTH^{WT} and its variants. 18
Supplementary Figure 20. Comparison of residues in the active sites of CtNTH and VaNTH. 18
Supplementary Figure 21. Representative binding poses of dGMP in CtNTH generated by docking. 19
Supplementary Figure 22. RMSD curves of three replicas for MD of CtNTH/dGMP. 19
Supplementary Figure 23. CtNTH/dGMP system: representative structures and statistical analysis of productive conformation for mode 1 and mode 2. 20
Supplementary Figure 24. Representative binding poses of dAMP in CtNTH generated by docking. 20

Supplementary Figure 25. RMSD curves of three replicas for MD of CtNTH/dAMP. 21
Supplementary Figure 26. CtNTH/dAMP system: representative structures and statistical analysis of mode 1 and mode 2......21
Supplementary Figure 27. Representative binding poses of dAMP and dGMP in AdaV. 22
Supplementary Figure 28. RMSD curves of three replicas for MD of AdaV/dAMP. ..22
Supplementary Figure 29. AdaV/dAMP system: representative structures and statistical analysis of mode 1 and mode 2......23
Supplementary Figure 30. RMSD curves of three replicas for MD of AdaV/dGMP. ..23
Supplementary Figure 31. AdaV/dGMP system: representative structures and statistical analysis of mode 1 and mode 2......24
Supplementary Figure 32. Hydrogen bond occurrence analysis for mode 2 of CtNTH/dGMP. 25
Supplementary Figure 33. HPLC analysis of enzymatic reactions catalyzed by I176N, V273A, H274Y, V301I, G304R and L305F variants of CtNTH with dGMP (1) and dAMP (3). 26
Supplementary Figure 34. HPLC analysis of enzymatic reactions catalyzed by H274Y, H274N and H274F variants of CtNTH with dAMP (3) and dGMP (1)......27
Supplementary Figure 35. HPLC analysis of enzymatic reactions catalyzed by I176N-H274Y, V273A-H274Y and H274Y-V301I variants of CtNTH with dAMP (3) and dGMP (1). 27
Supplementary Figure 36. Steady-state kinetic analysis for CtNTH^{H274Y} with dGMP and dAMP as substrate......28
Supplementary Figure 37. HPLC analysis of enzymatic reactions catalyzed by N175I, A272V, Y273H, I299V, G302R and F303L variants of AdaV with dGMP (1) and dAMP (3). 29
Supplementary Figure 38. HPLC analysis of enzymatic reactions catalyzed by A272V-Y273H, R302G-F303V and R302G-F303L variants of AdaV with dGMP (1) and dAMP (3). 30
Supplementary Figure 39. HPLC analysis of enzymatic reactions catalyzed by F303L, F303A, F303V, F303I, Y273F and Y273N variants of AdaV with dGMP (1) and dAMP (3). 31
Supplementary Figure 40. Steady-state kinetic analysis for AdaV^{F303V} with dGMP and dAMP as substrate. 32
Supplementary Figure 41. RMSD curves of three replicas for MD of CtNTH^{H274Y}/dGMP. 33
Supplementary Figure 42. RMSD curves of three replicas for MD of CtNTH^{H274Y}/dAMP. 33
Supplementary Figure 43. CtNTH^{H274Y}/dGMP system: representative structures and statistical analysis of mode 1 and mode 2......34
Supplementary Figure 44. CtNTH^{H274Y}/dAMP system: representative structures and statistical analysis of mode 1 and mode 2......35
Supplementary Figure 45. (a) Relative halogenation activities of CtNTH^{WT} and

CtNTH^{R238A} to dGMP. (b) Relative halogenation activities of CtNTH^{H274Y} and CtNTH^{H274Y_R238A} to dAMP. 36
Supplementary Figure 46. RMSD curves of three replicas for MD of AdaV^{F303V}/dGMP. 36
Supplementary Figure 47. RMSD curves of three replicas for MD of AdaV^{F303V}/dAMP. 37
Supplementary Figure 48. AdaV^{F303V}/dGMP system: representative structures and statistical analysis of mode 1 and mode 2....................37
Supplementary Figure 49. AdaV^{F303V}/dAMP system: representative structures and statistical analysis of mode 1 and mode 2....................38
Supplementary Figure 50. Dynamic cross-correlation analysis of CtNTH and CtNTH^{H274Y}....................39
Supplementary Figure 51. Dynamic cross-correlation analysis of AdaV and AdaV^{F303V}. 40

List of supplementary tables:

Supplementary Table 1. Percent identity matrix of AdaV analogs. 41
Supplementary Table 2. Average distances of O···H and C2′···Cl in mode 1 and mode 2 for CtNTH/dGMP and AdaV/dAMP systems. 41
Supplementary Table 3. Statistical analysis of substrate binding modes and productive conformations. 42
Supplementary Table 4. Statistical analysis of substrate binding modes and productive conformations in MD simulations of $CtNTH^{H274Y}$ and $AdaV^{F303V}$ with dGMP and dAMP. 42
Supplementary Table 5. Hydrogen bond occurrence between R302 and the phosphate group of the substrates in AdaV and $AdaV^{F303V}$ systems....................................43
Supplementary Table 6. Correlation coefficient of residue 274 with F272 and substrates in CtNTH and $CtNTH^{H274Y}$ systems. 43
Supplementary Table 7. Correlation coefficient of R302 with the phosphate group of dAMP/dGMP in AdaV and $AdaV^{F303V}$ systems..43
Supplementary Table S8. Harmonic restraints in MD simulations.............................43

1. Supplementary Figures

1.1 Identification of new dGMP nucleotide halogenases – Figure S1 to S18

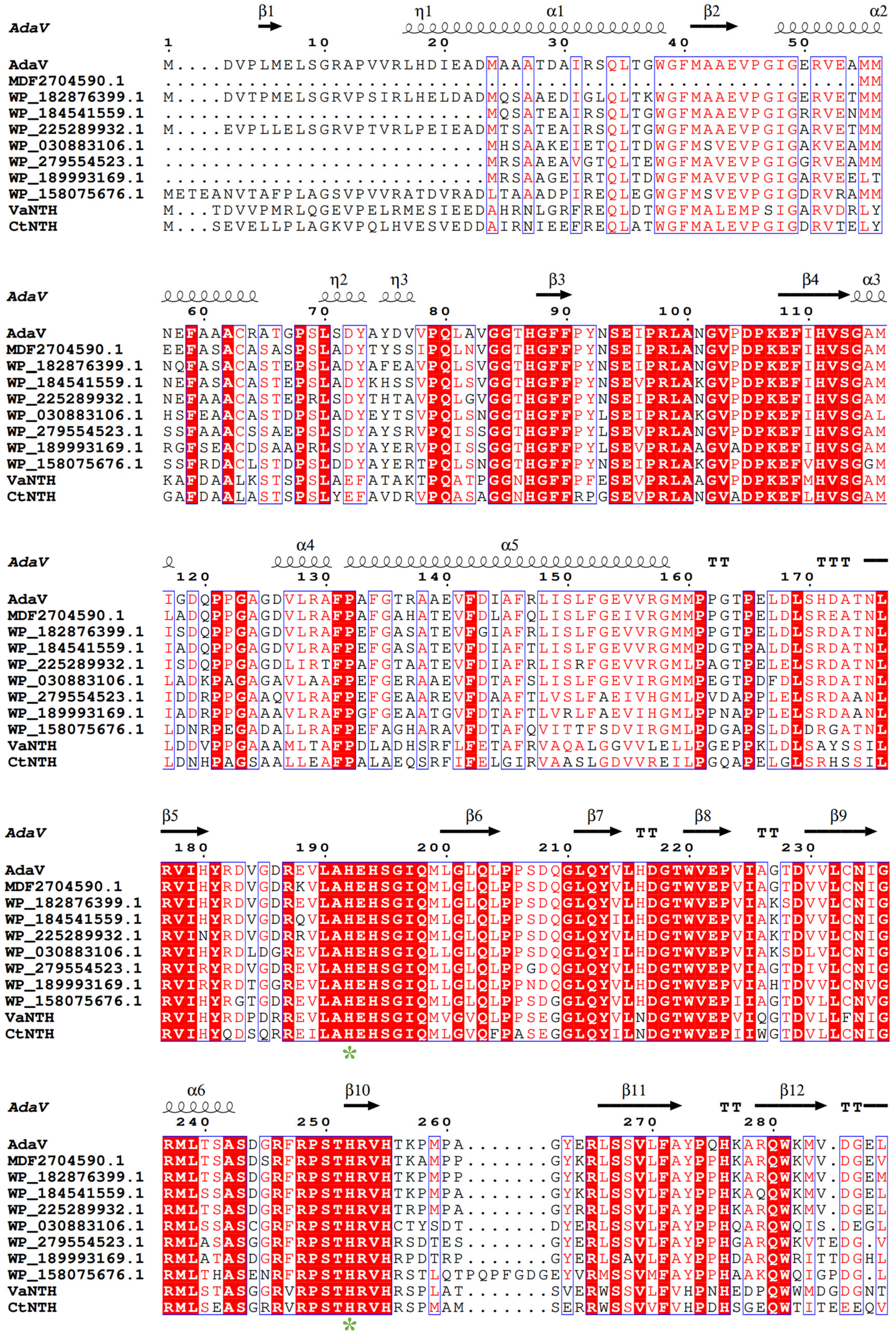

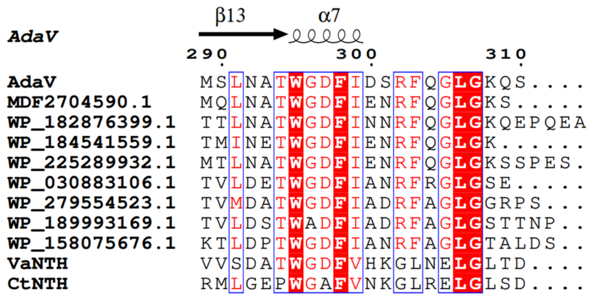

Supplementary Figure 1. Sequence alignment of VaNTH and CtNTH with AdaV analogs.

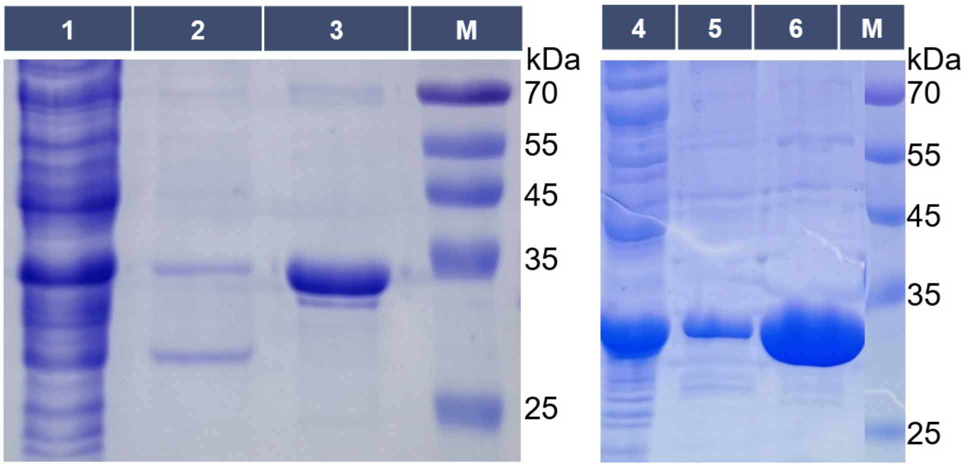

Supplementary Figure 2. SDS-PAGE analysis of VaNTH and CtNTH. 1, The supernatant of cell lysate contained VaNTH; **2**, The precipitate of cell lysate containing VaNTH; **3**, Purified VaNTH; **4**, The supernatant of cell lysate containing CtNTH; **5**, The precipitate of cell lysate containing CtNTH; **6**, Purified CtNTH; M: Molecular weight marker.

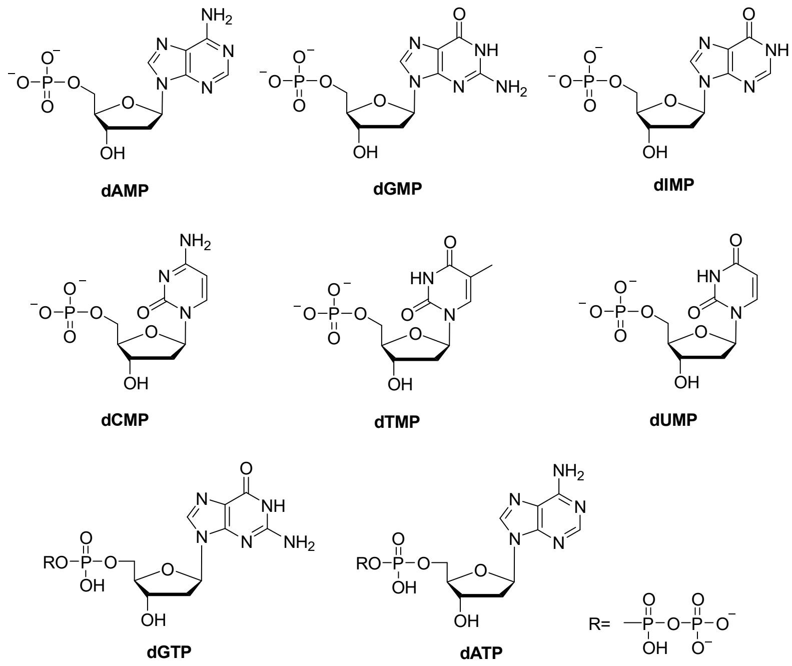

Supplementary Figure 3. Substrate panel of natural nucleotides and nucleoside triphosphates.

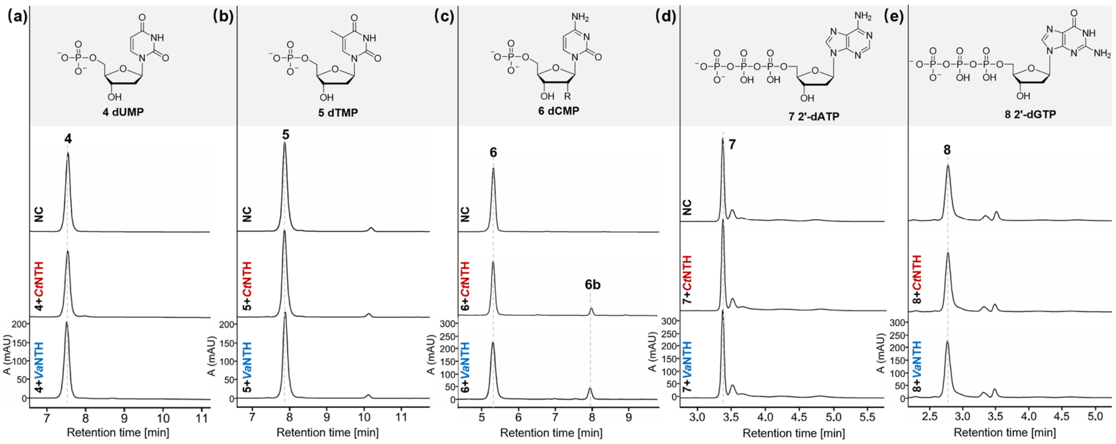

Supplementary Figure 4. HPLC analysis of enzymatic reactions catalyzed by CtNTH with substrates 4-8. No corresponding chlorination products were detected. 6b was determined to be 2'-deoxyuridine by LC-HRMS. All HPLC spectra are representative of at least three experimental replicates.

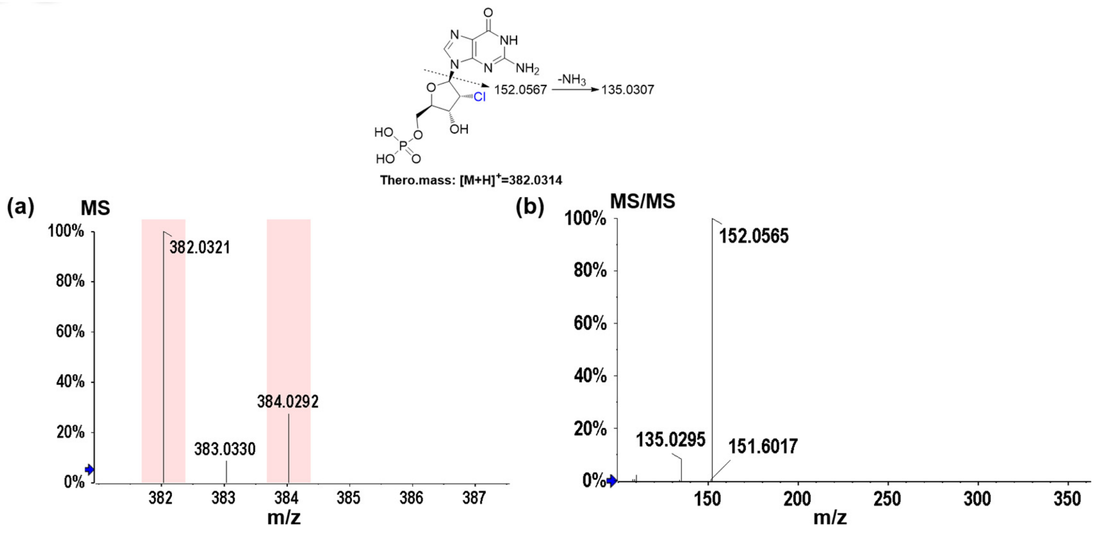

Supplementary Figure 5. LC-HRMS analysis of the chlorination products 2′-Cl-dGMP. (a) LC-HRMS analysis of **1a**. (b) LC-HRMS/MS analysis of **1a**.

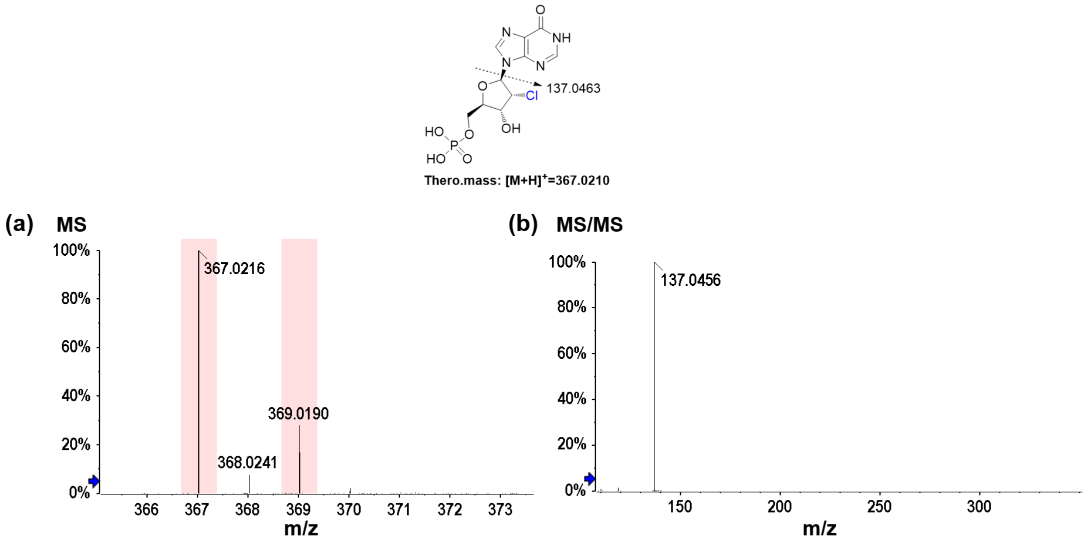

Supplementary Figure 6. LC-HRMS analysis of the chlorination products 2′-Cl-dIMP. (a) LC-HRMS analysis of **2a**. (b) LC-HRMS/MS analysis of **2a**.

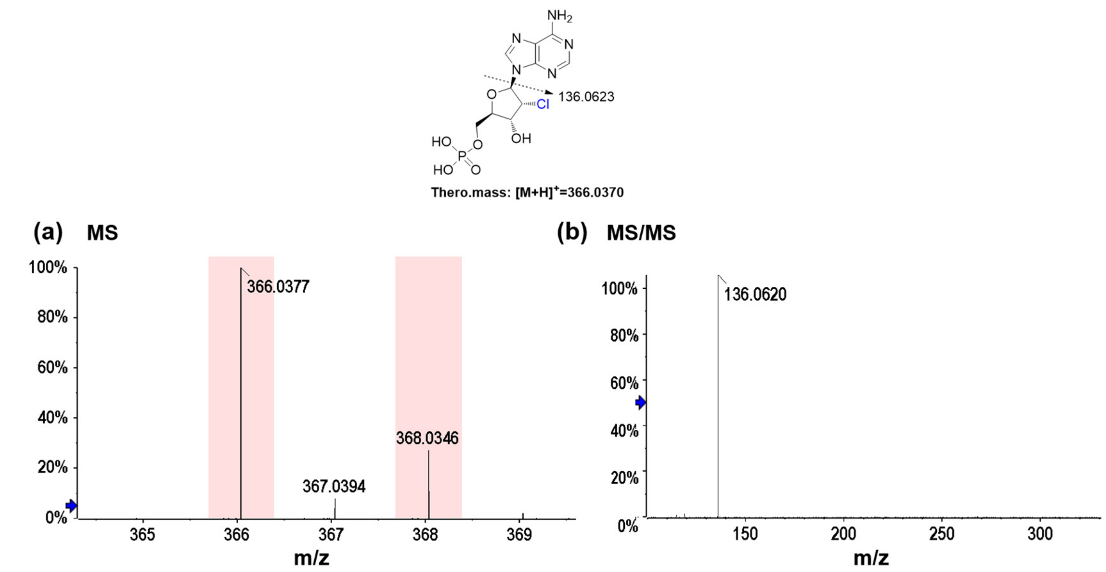

Supplementary Figure 7. LC-HRMS analysis of the chlorination products 2′-Cl-dAMP. (a) LC-HRMS analysis of **3a**. (b) LC-HRMS/MS analysis of **3a**.

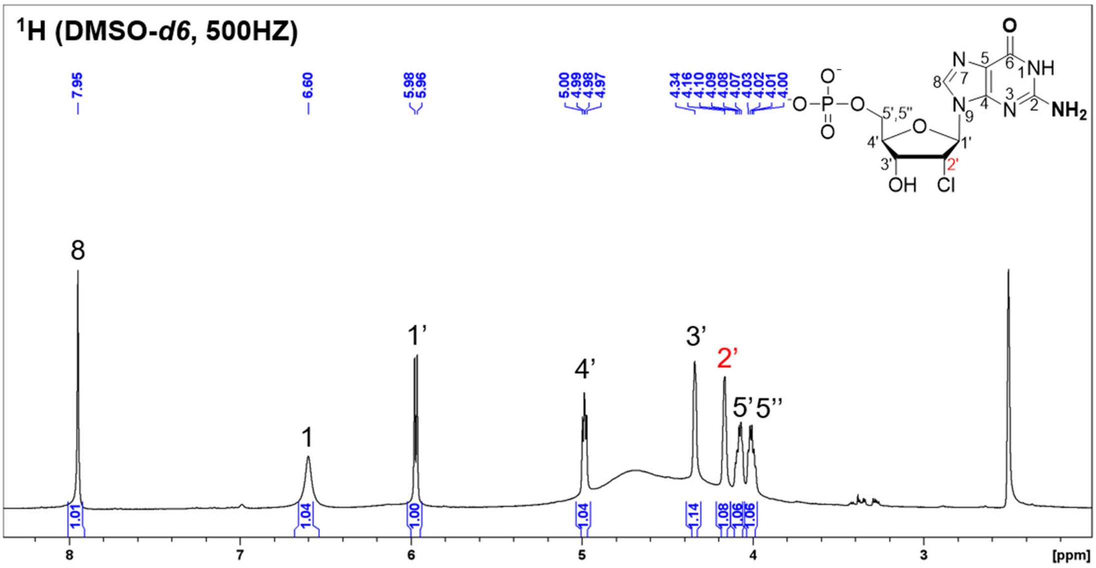

Supplementary Figure 8. ^{1}H NMR spectrum of 1a (2′-Cl-dGMP) (500MHz, DMSO-*d6*).

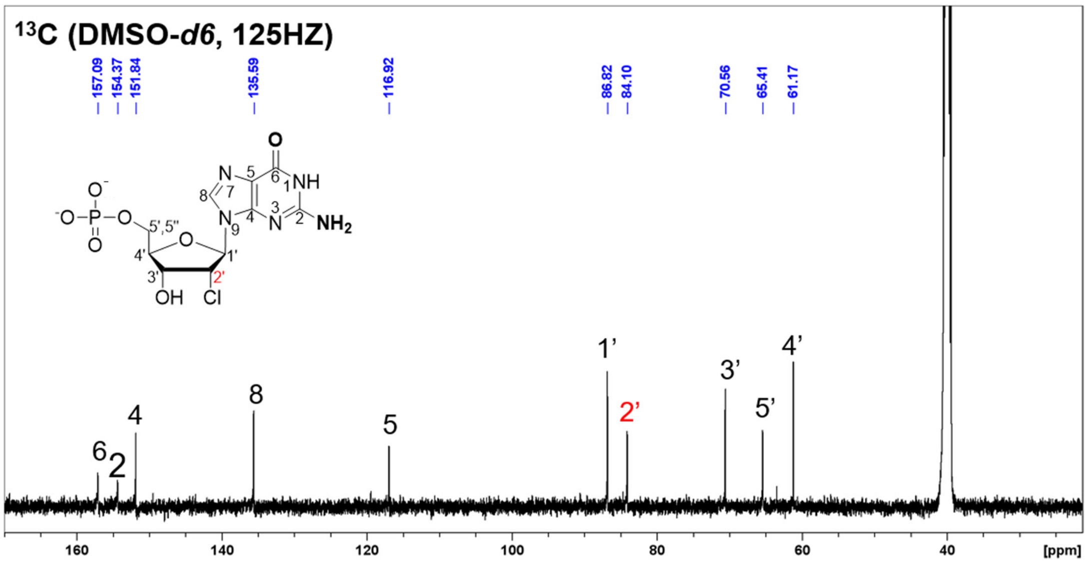

Supplementary Figure 9. ^{13}**C NMR spectrum of 1a (2′-Cl-dGMP) (125MHz, DMSO-*d6*).**

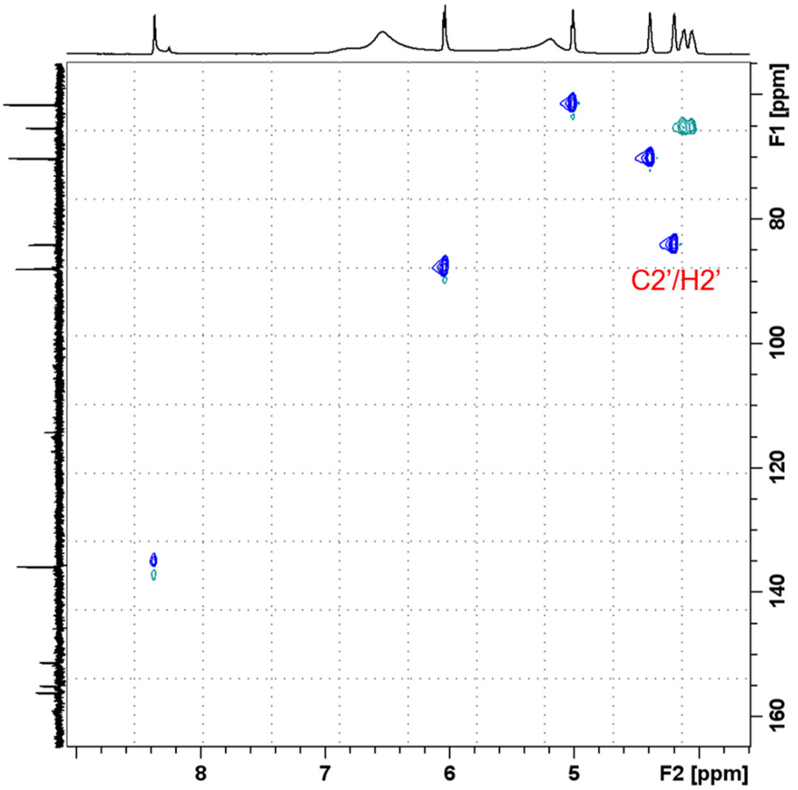

Supplementary Figure 10. HSQC spectrum of 1a (2′-Cl-dGMP).

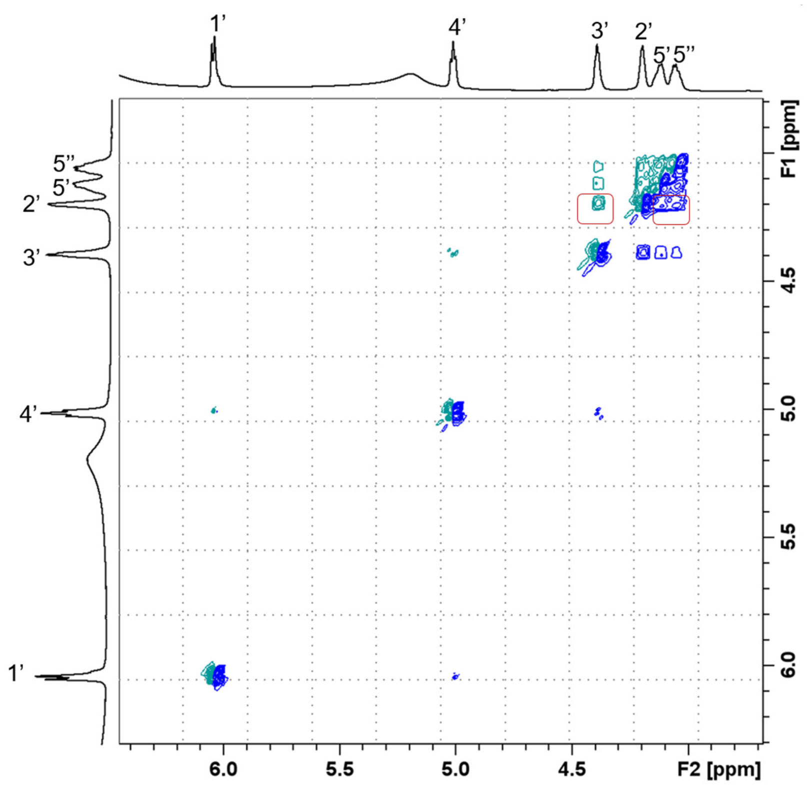

Supplementary Figure 11. NOESY spectrum of 1a (2′-Cl-dGMP).

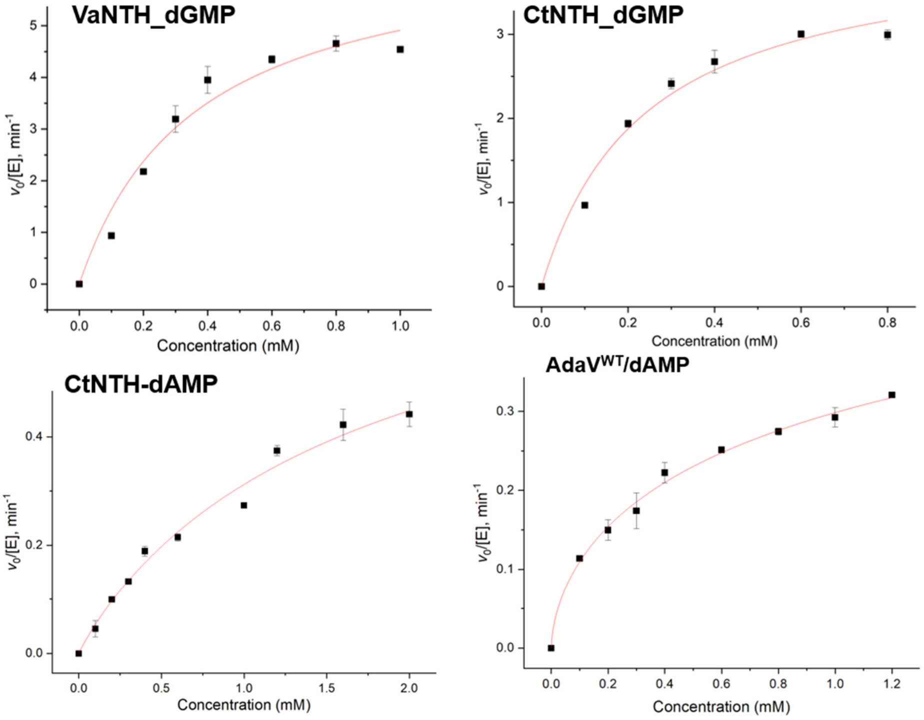

Kinetic Parameter	VaNTH_dGMP	CtNTH_dGMP	CtNTH_dAMP	AdaV_dAMP
k_{cat} (min^{-1})	6.7 ± 0.3	4.1 ± 0.2	0.55 ± 0.07	0.69 ± 0.03
K_m (mM)	0.36 ± 0.04	0.23 ± 0.04	0.63 ± 0.1	1.56 ± 0.2
k_{cat}/K_m ($mM^{-1}min^{-1}$)	18.6 ± 0.4	17.8 ± 0.3	0.87 ± 0.09	0.44 ± 0.1

Supplementary Figure 12. Steady-state kinetic analysis for VaNTH, CtNTH, and AdaV with dGMP and dAMP substrates. The table contains k_{cat}, K_m, and k_{cat}/K_m calculated by non-linear curve fitting to the Michaelis-Menten equation. Data are presented as mean ± s.d. (n = 3 biologically independent experiments).

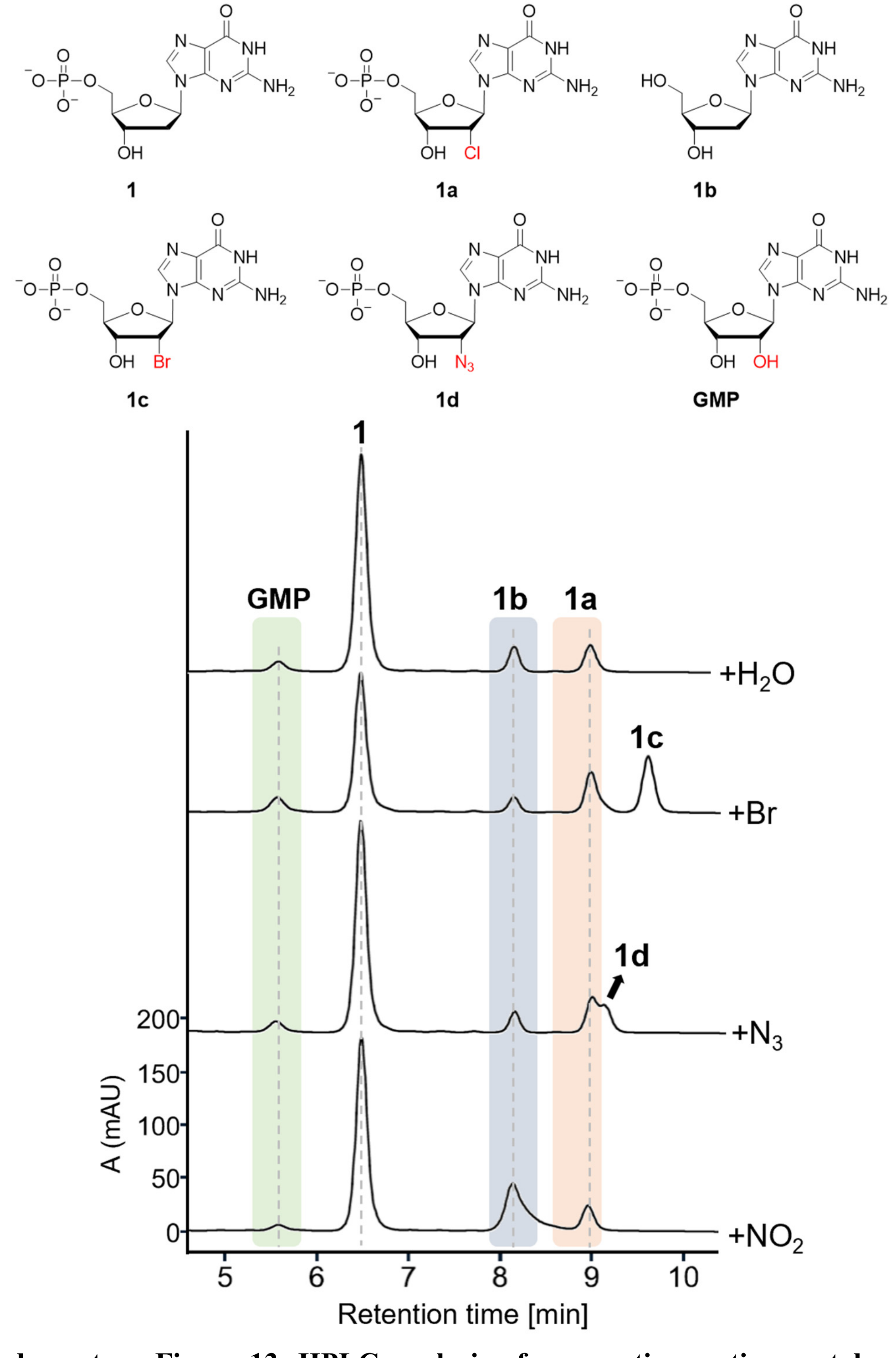

Supplementary Figure 13. HPLC analysis of enzymatic reactions catalyzed by CtNTH with dGMP and NaBr/NaI/NaN_3/$NaNO_2$. 2′-Cl-dGMP was also produced in the assay due to the presence of trace Cl^- from the enzyme storage buffer. All HPLC spectra are representative of at least three experimental replicates.

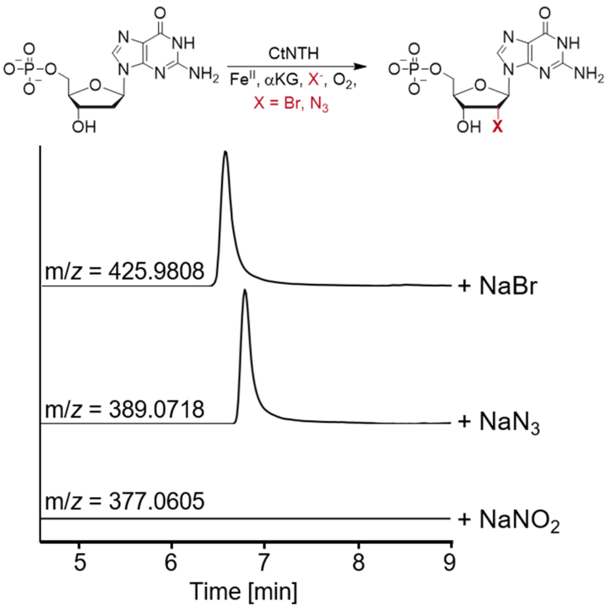

Supplementary Figure 14. LC-HRMS analysis of enzymatic reactions catalyzed by CtNTH with dGMP and NaBr/NaN_3/$NaNO_2$.

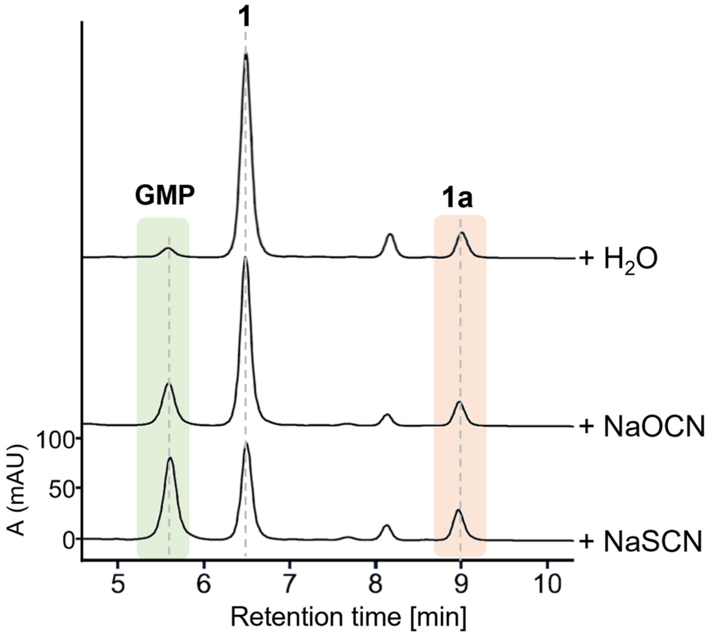

Supplementary Figure 15. HPLC analysis of enzymatic reactions catalyzed by CtNTH with dGMP and NaOCN/NaSCN. 2′-Cl-dGMP were also produced in the assay due to the presence of trace Cl^- from the enzyme storage buffer. All HPLC spectra are representative of at least three experimental replicates.

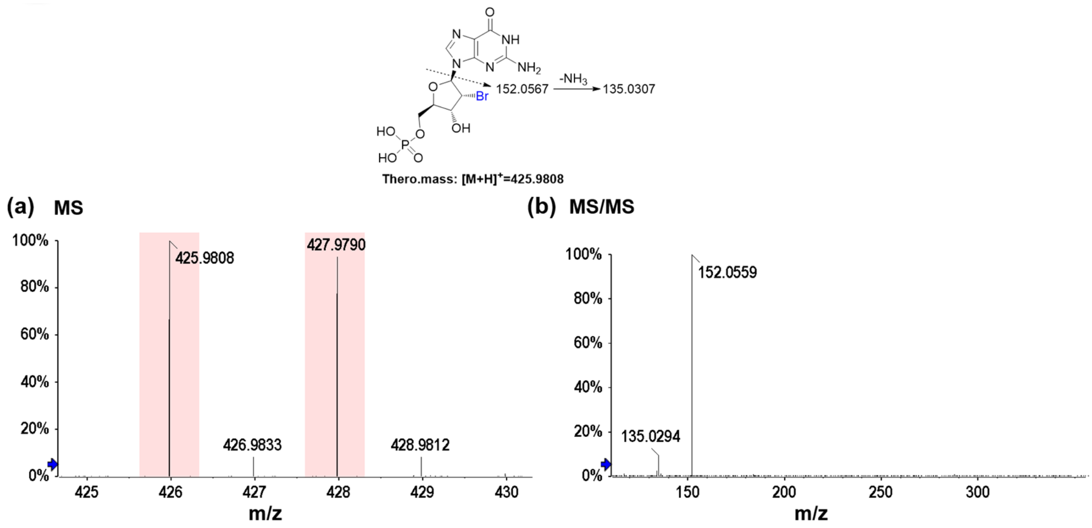

Supplementary Figure 16. LC-HRMS analysis of the bromination products 2′-Br-dGMP. (a) LC-HRMS analysis of 2′-Br-dGMP. (b) LC-HRMS/MS analysis of 2′-Br-dGMP.

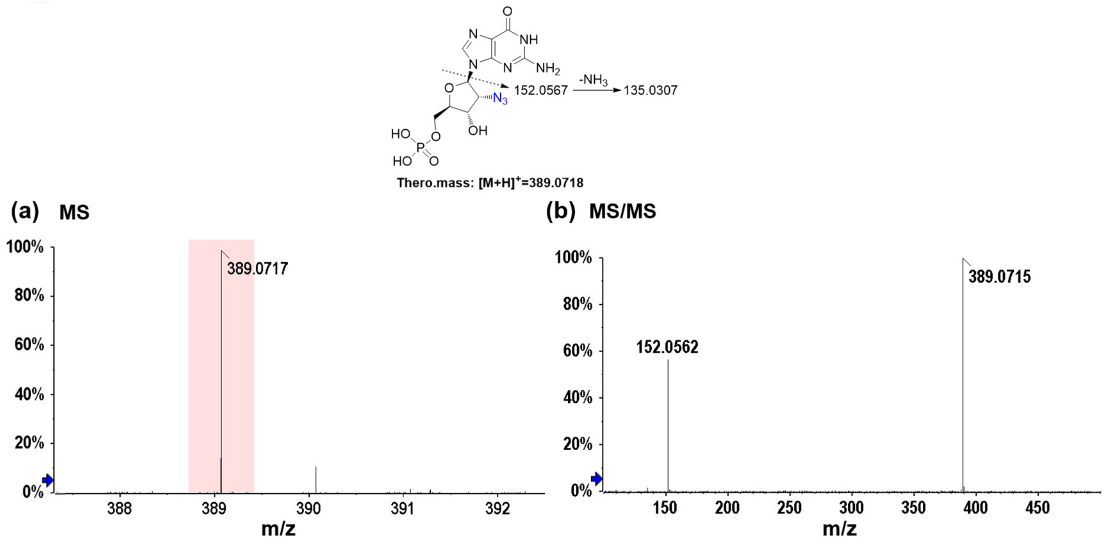

Supplementary Figure 17. LC-HRMS analysis of the azidation product 2′-N_3-dGMP. (a) LC-HRMS analysis of 2′-N_3-dGMP. (b) LC-HRMS/MS analysis of 2′-N_3-dGMP.

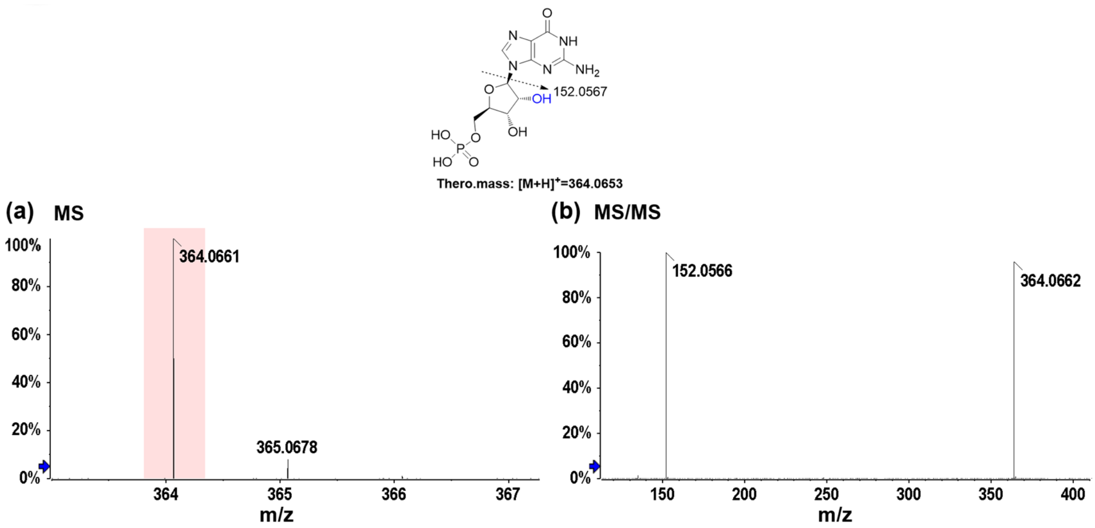

Supplementary Figure 18. LC-HRMS analysis of the hydroxylation product GMP. (a) LC-HRMS analysis of GMP. (b) LC-HRMS/MS analysis of GMP.

1.2 Binding mode and mechanism of nucleotide specificity – Figure S19 to S32

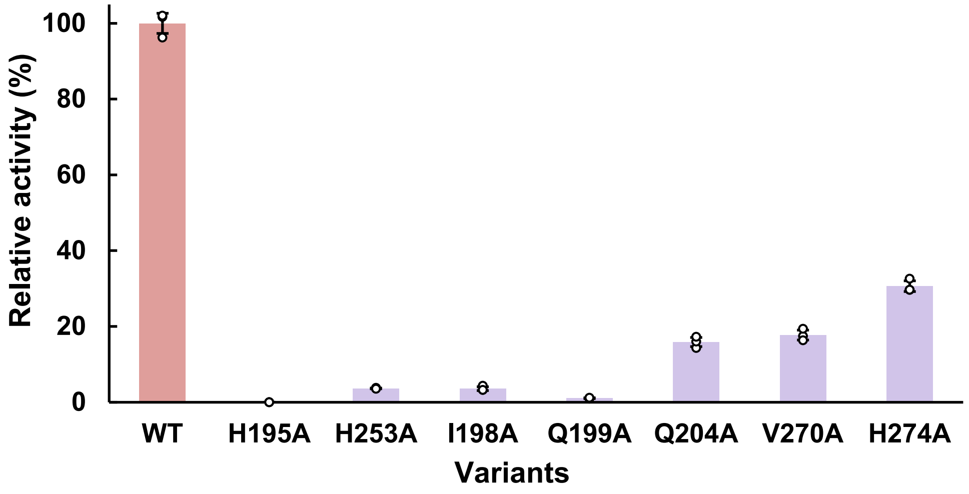

Supplementary Figure 19. Relative activities of CtNTH^{WT} and its variants. Data are presented as mean ± s.d. (n = 3 biologically independent experiments).

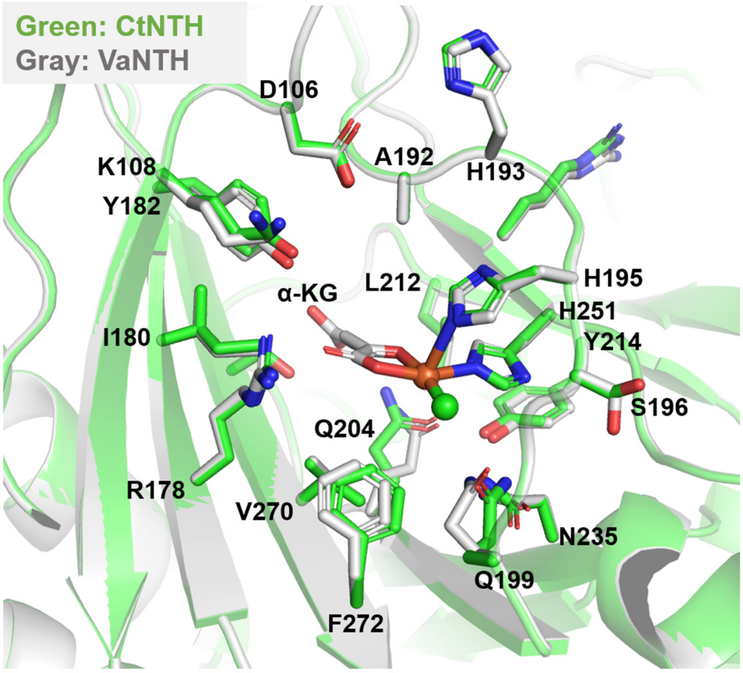

Supplementary Figure 20. Comparison of residues in the active sites of CtNTH and VaNTH.

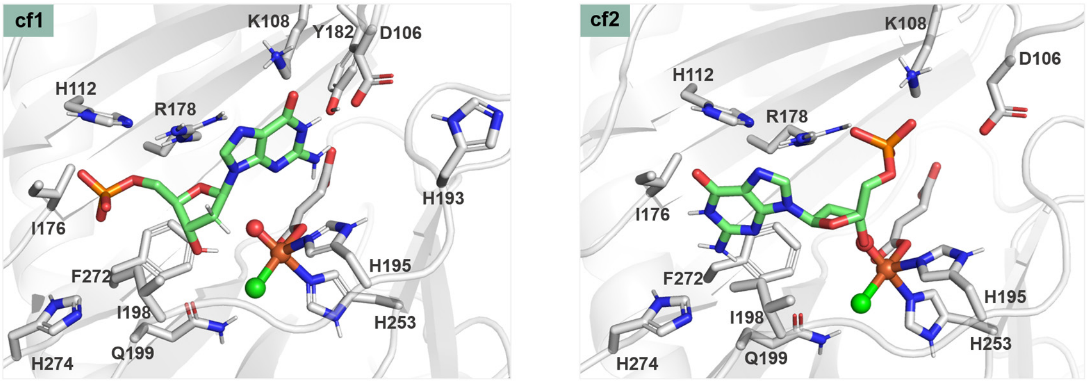

Supplementary Figure 21. Representative binding poses of dGMP in CtNTH generated by docking.
Two representative binding poses (cf1 and cf2) were obtained by docking dGMP to the active site of CtNTH. cf1 is similar to the binding pose observed in the crystal structure of AdaV/dAMP[2]. It is also consistent with the mutagenesis experiment where D106A totally abolish all chlorination activity. In contrast, cf2 is not consistent with the D106A mutagenesis experiment as there is no interaction between D106 and dGMP in cf2.

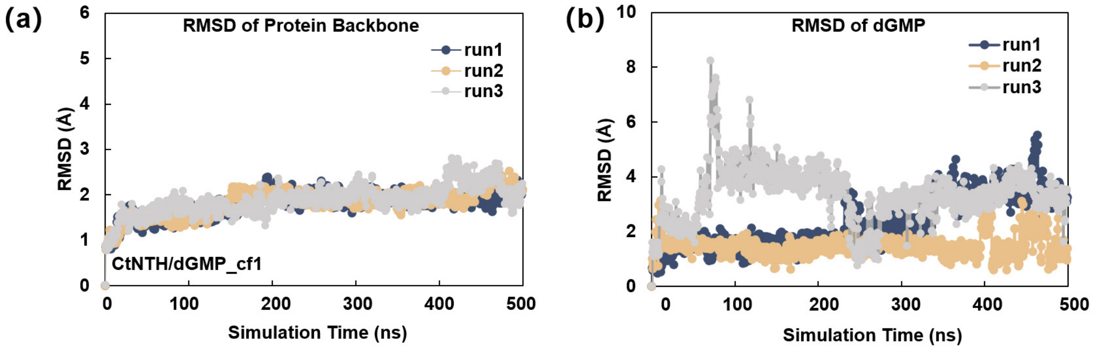

Supplementary Figure 22. RMSD curves of three replicas for MD of CtNTH/dGMP. (a) RMSD of the protein backbone calculated relative to the first frame; (b) RMSD of dGMP calculated relative to the first frame. RMSD: root mean squared deviation.

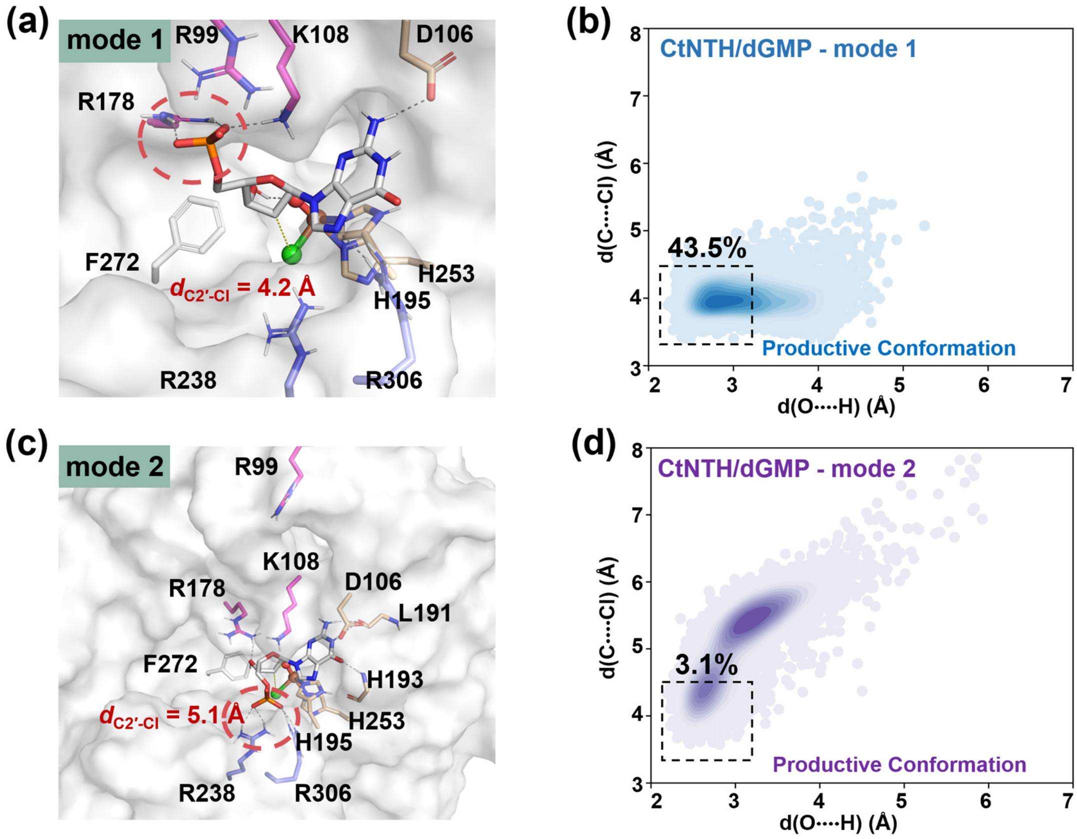

Supplementary Figure 23. CtNTH/dGMP system: representative structures and statistical analysis of productive conformation for mode 1 and mode 2. Representative structures of mode 1 (a) and mode 2 (c). Distance distributions of C2′···Cl and O···H (in Å) in mode 1 (b) and mode 2 (d) of CtNTH/dGMP. Two-dimensional plot of discrete points with Gaussian kernel density estimation of the distributions. The numbers in (b) and (d) are the percentage of productive conformations of each mode in the total frames. The productive conformations ($d_{O\text{-}H} \leq$ 3.2 Å, $d_{C\text{-}Cl} \leq$ 4.5 Å) are indicated with a dotted box.

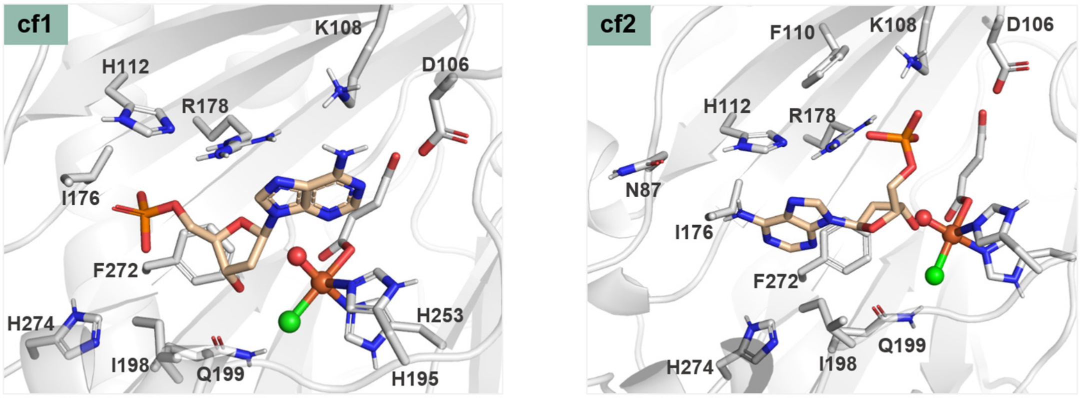

Supplementary Figure 24. Representative binding poses of dAMP in CtNTH generated by docking.

Two representative binding poses (cf1 and cf2) were obtained by docking dAMP to the

active stie of CtNTH. The top-ranked poses (cf1), which is similar to the binding pose in the crystal structure of AdaV/dAMP[2], was used as the starting structure for further MD simulation.

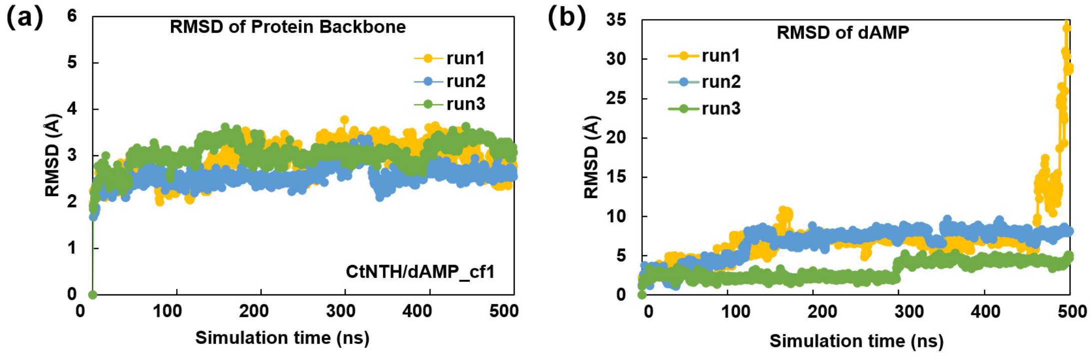

Supplementary Figure 25. RMSD curves of three replicas for MD of CtNTH/dAMP. (a) RMSD of the protein backbone calculated relative to the first frame; (b) RMSD of dAMP calculated relative to the first frame.

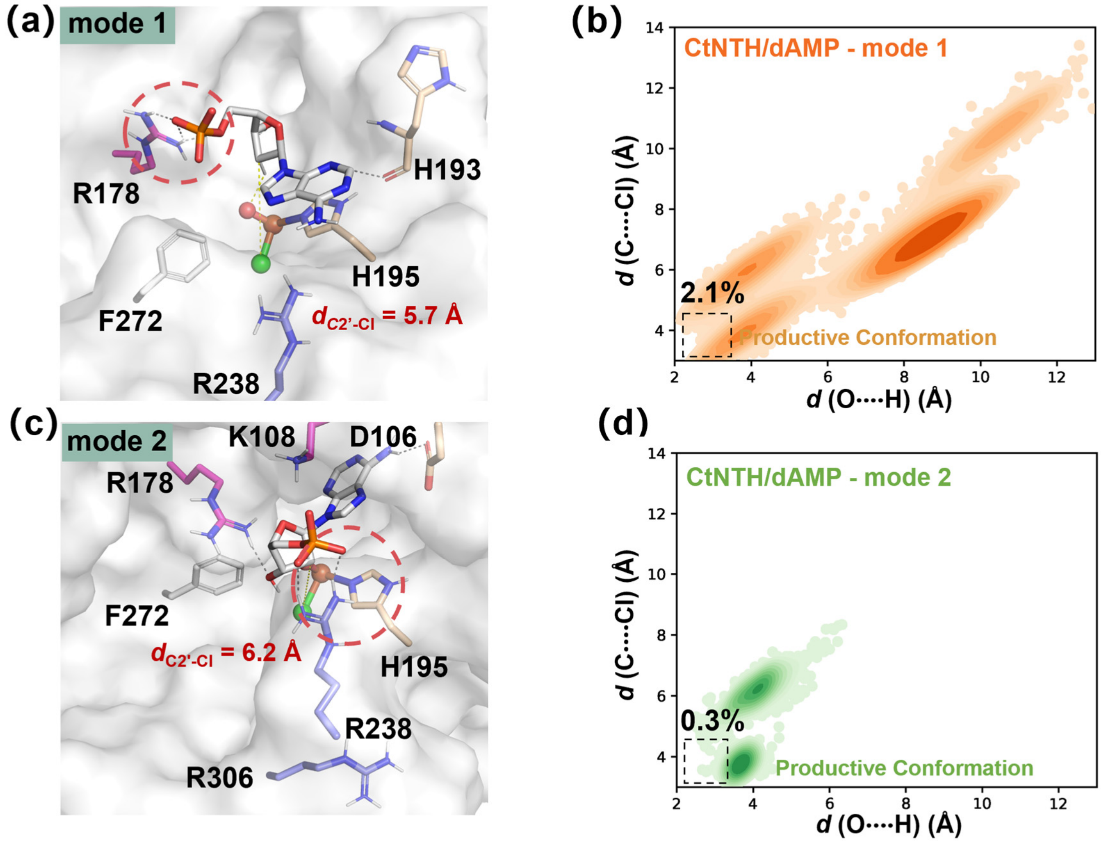

Supplementary Figure 26. CtNTH/dAMP system: representative structures and statistical analysis of mode 1 and mode 2. Representative structures of mode 1 (a) and mode 2 (c). Distance distributions of C2′···Cl and O···H (in Å) in mode 1 (b) and mode 2 (d) of CtNTH/dAMP. Two-dimensional plot of discrete points with Gaussian kernel density estimation of the distributions. The numbers in (b) and (d) represent the percentage of productive conformations of each mode in total frames. The productive conformations ($d_{O\text{-}H} \leq 3.2$ Å, $d_{C\text{-}Cl} \leq 4.5$ Å) are indicated with a dotted box.

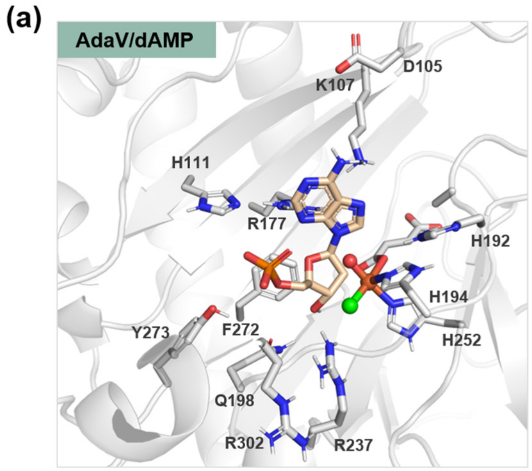

Supplementary Figure 27. Representative binding poses of dAMP and dGMP in AdaV. The binding conformation of AdaV/dAMP (a) was obtained from the reported crystal structure[2], the binding conformation of AdaV/dGMP (b) was generated by docking.

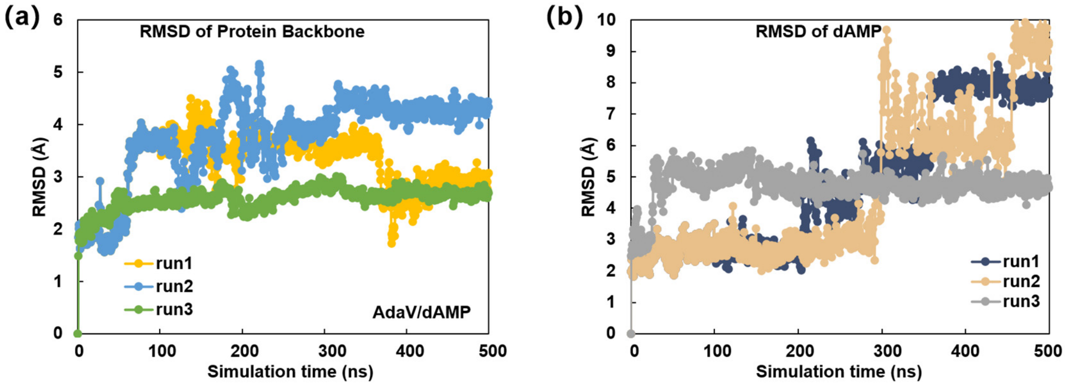

Supplementary Figure 28. RMSD curves of three replicas for MD of AdaV/dAMP. (a) RMSD of the protein backbone calculated relative to the first frame; (b) RMSD of dAMP calculated relative to the first frame.

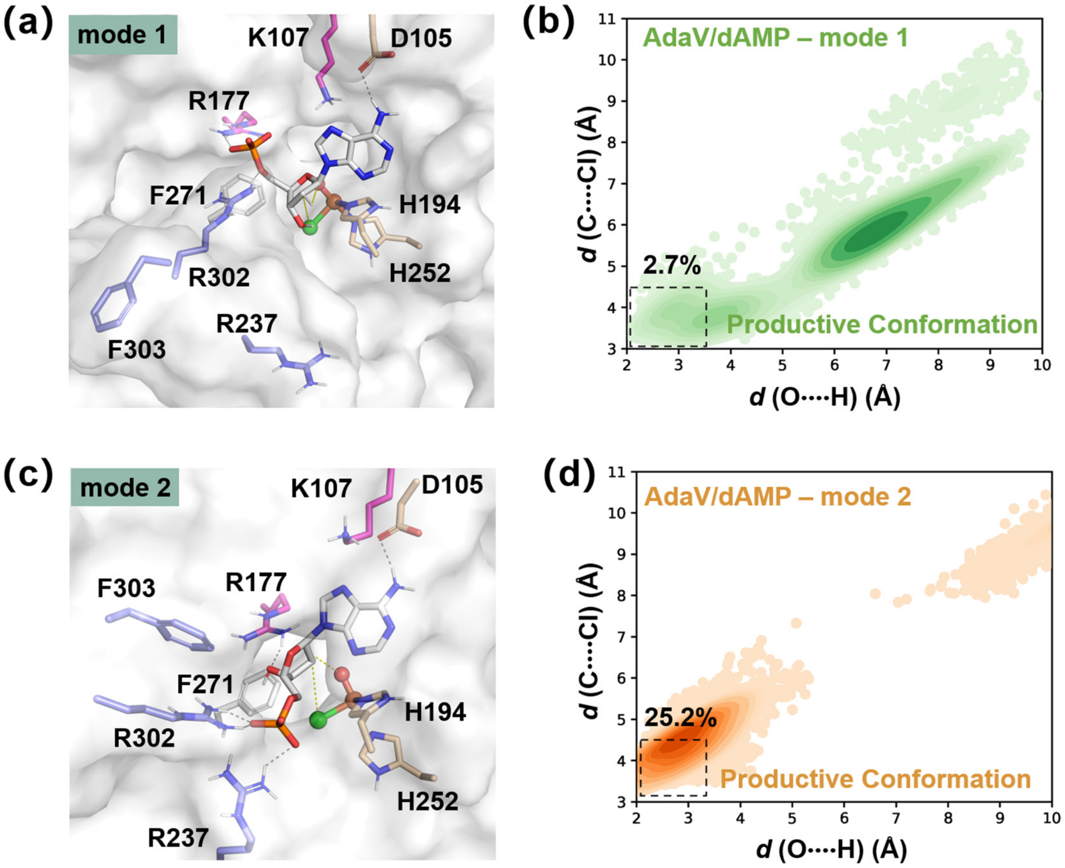

Supplementary Figure 29. AdaV/dAMP system: representative structures and statistical analysis of mode 1 and mode 2. Representative structures of mode 1 (a) and mode 2 (c). Distance distributions of C2′···Cl and O···H (in Å) in mode 1 (b) and mode 2 (d) of AdaV/dAMP. Two-dimensional plot of discrete points with Gaussian kernel density estimation of the distributions. The numbers in (b) and (d) donate the percentage of productive conformations of each mode in total frames. The productive conformations ($d_{O\text{-}H} \leq 3.2$ Å, $d_{C\text{-}Cl} \leq 4.5$ Å) are indicated with a dotted box.

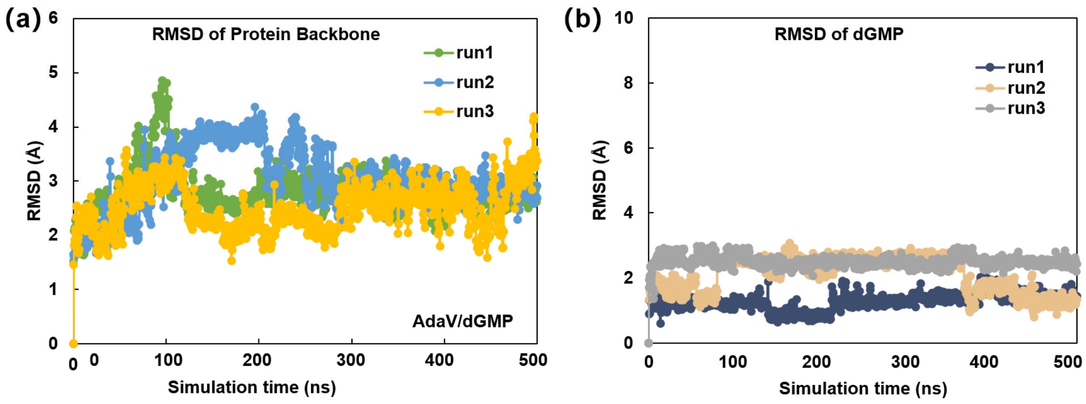

Supplementary Figure 30. RMSD curves of three replicas for MD of AdaV/dGMP. (a) RMSD of the protein backbone calculated relative to the first frame; (b) RMSD of dGMP calculated relative to the first frame.

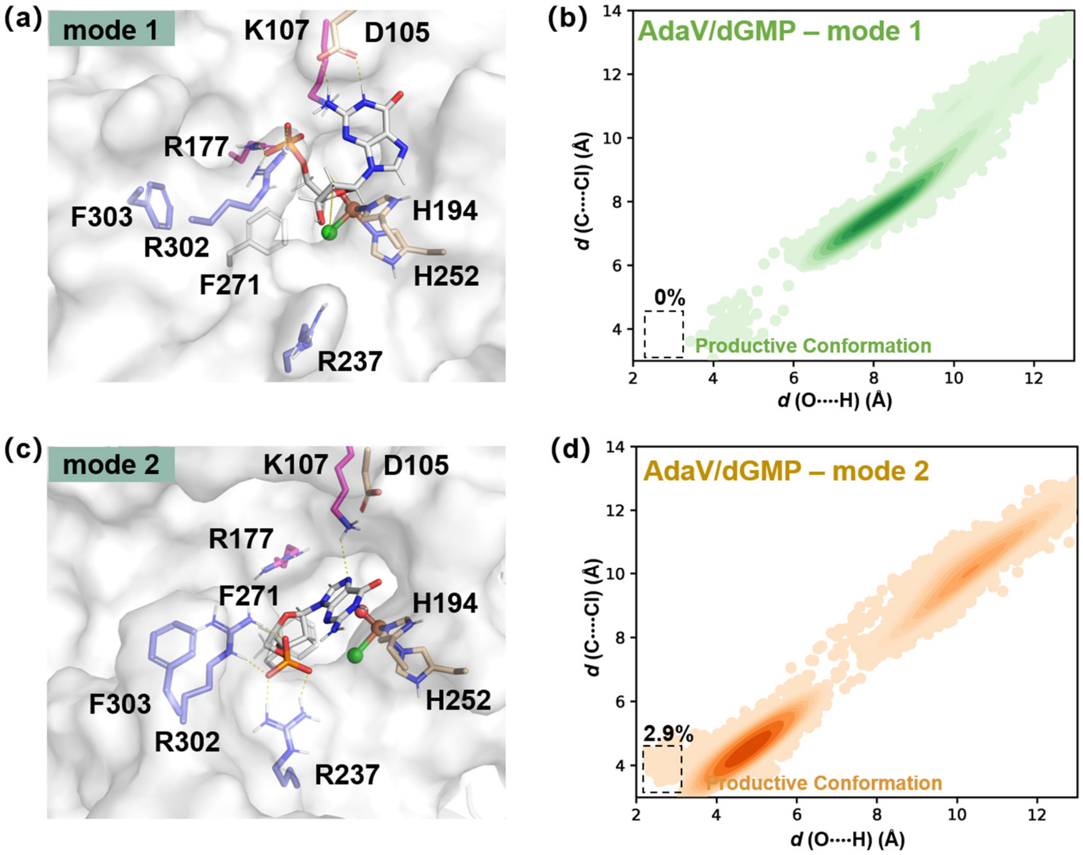

Supplementary Figure 31. AdaV/dGMP system: representative structures and statistical analysis of mode 1 and mode 2. Representative structures of mode 1 (a) and mode 2 (c). Distance distributions of C2′···Cl and O···H (in Å) in mode 1 (b) and mode 2 (d) of AdaV/dGMP. Two-dimensional plot of discrete points with Gaussian kernel density estimation of the distributions. The numbers in (b) and (d) donate the percentage of productive conformations of each mode in total frames. The productive conformations ($d_{O\text{-}H} \leq 3.2$ Å, $d_{C\text{-}Cl} \leq 4.5$ Å) are indicated with a dotted box.

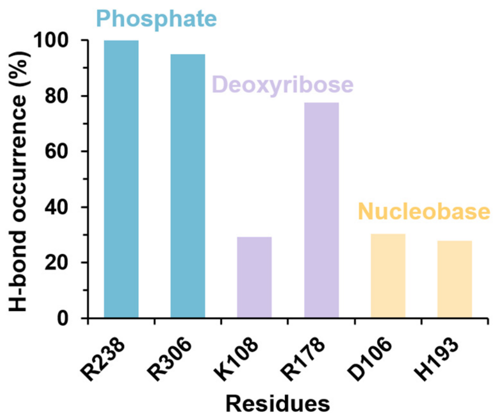

Supplementary Figure 32. Hydrogen bond occurrence analysis for mode 2 of CtNTH/dGMP. R238 and R306 form hydrogen bonds with the phosphate group of dGMP, K108 and R178 form hydrogen bonds with the deoxyribose, D106 and H193 form hydrogen bonds with the nucleobase moiety.

1.3 Engineering of nucleotide specificity– Figure S33 to S40

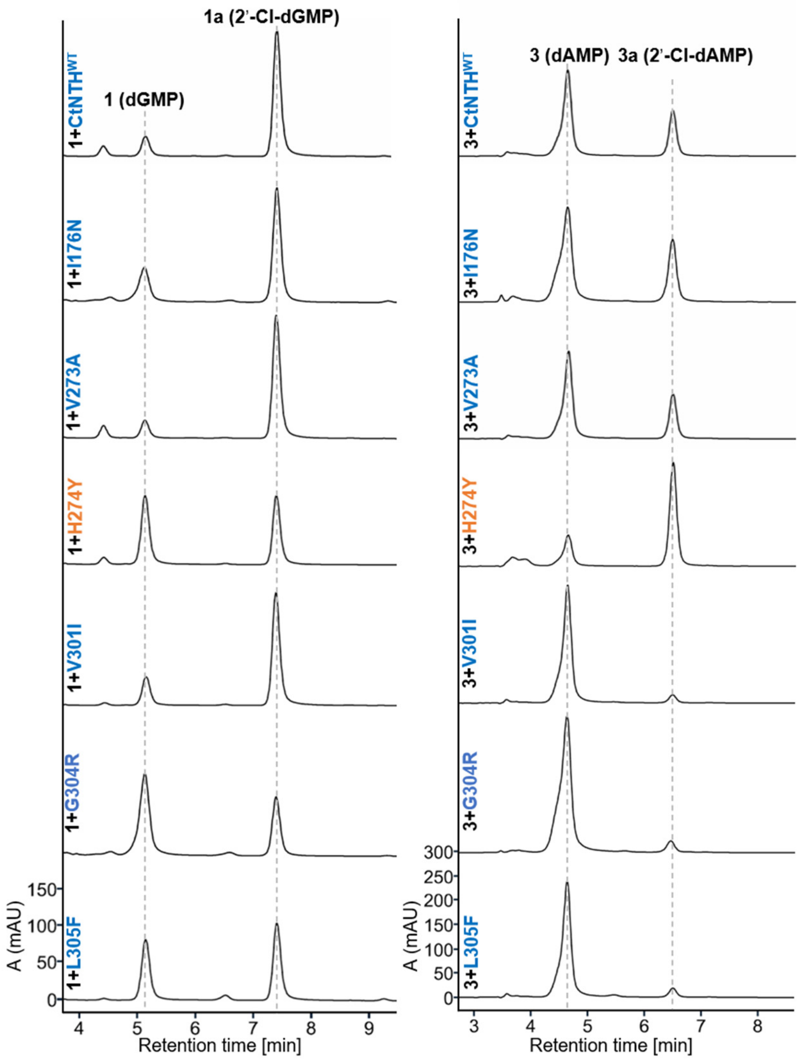

Supplementary Figure 33. HPLC analysis of enzymatic reactions catalyzed by I176N, V273A, H274Y, V301I, G304R and L305F variants of CtNTH with dGMP (1) and dAMP (3). All HPLC spectra are representative of at least three experimental replicates.

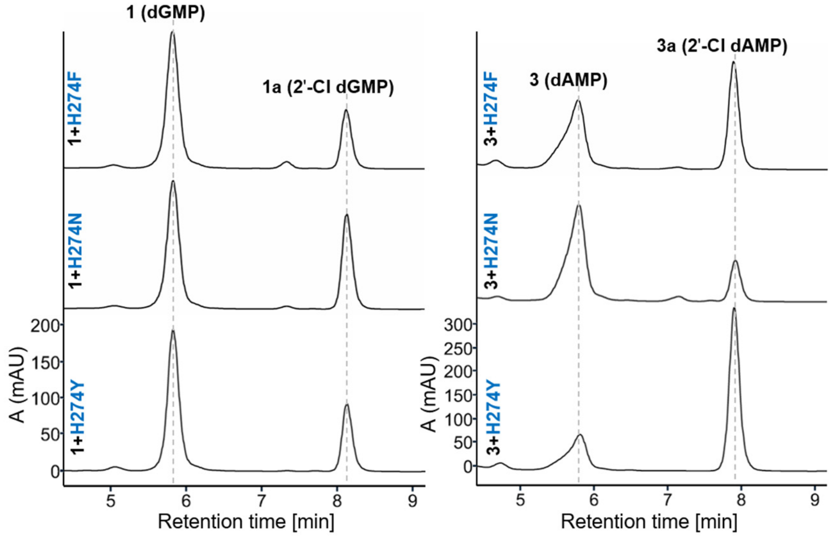

Supplementary Figure 34. HPLC analysis of enzymatic reactions catalyzed by H274Y, H274N and H274F variants of CtNTH with dAMP (3) and dGMP (1). All HPLC spectra are representative of at least three experimental replicates.

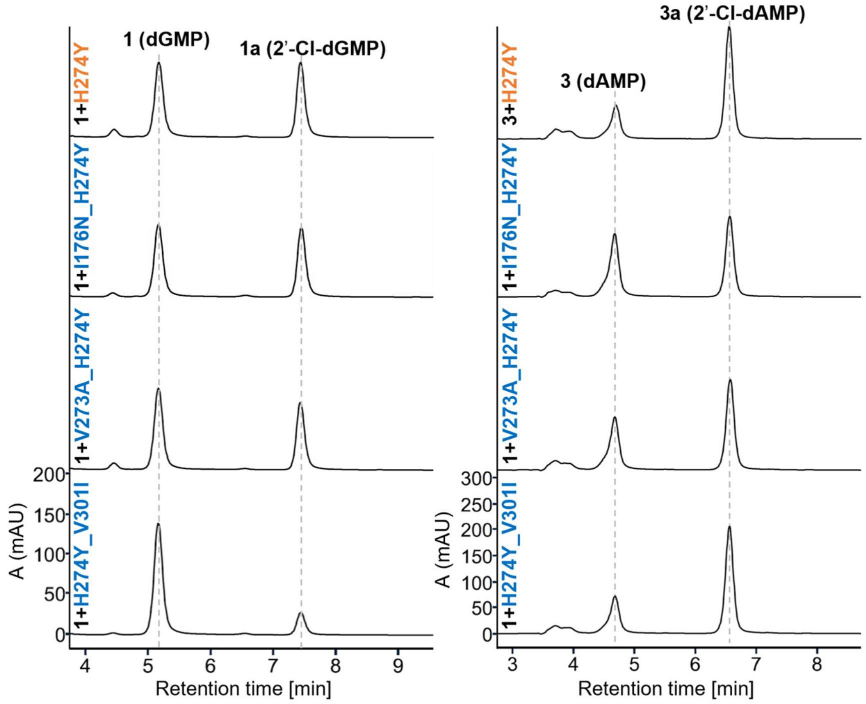

Supplementary Figure 35. HPLC analysis of enzymatic reactions catalyzed by I176N-H274Y, V273A-H274Y and H274Y-V301I variants of CtNTH with dAMP (3) and dGMP (1). All HPLC spectra are representative of at least three experimental replicates.

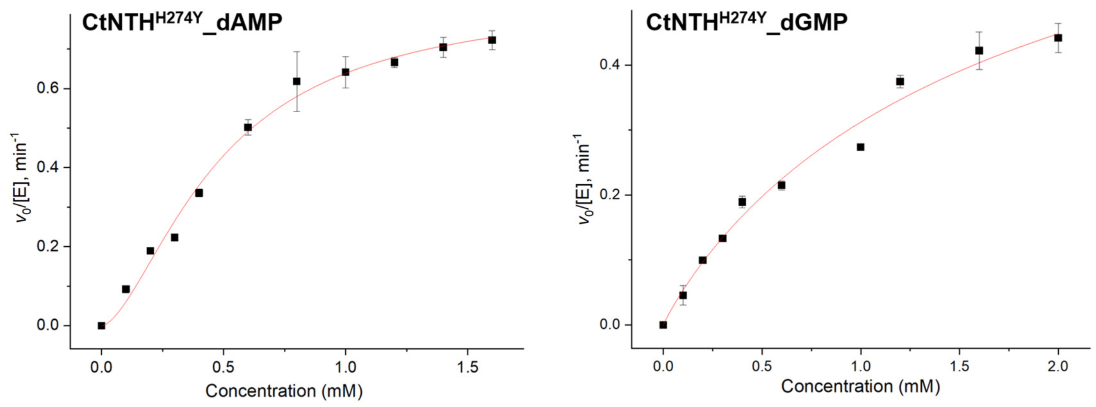

Kinetic Parameter	CtNTH^{H274Y}_dAMP	CtNTH^{H274Y}_dGMP
k_{cat} (min^{-1})	0.83 ± 0.1	0.86 ± 0.09
K_m (mM)	0.48 ± 0.06	1.83 ± 0.2
k_{cat}/K_m (mM^{-1}min^{-1})	1.83 ± 0.06	0.47 ± 0.08

Supplementary Figure 36. Steady-state kinetic analysis for CtNTH^{H274Y} with dGMP and dAMP as substrate. The table contains k_{cat}, K_m, and k_{cat}/K_m calculated by non-linear curve fitting to the Michaelis-Menten equation. Data are mean ± s.d. (n = 3 biologically independent experiments).

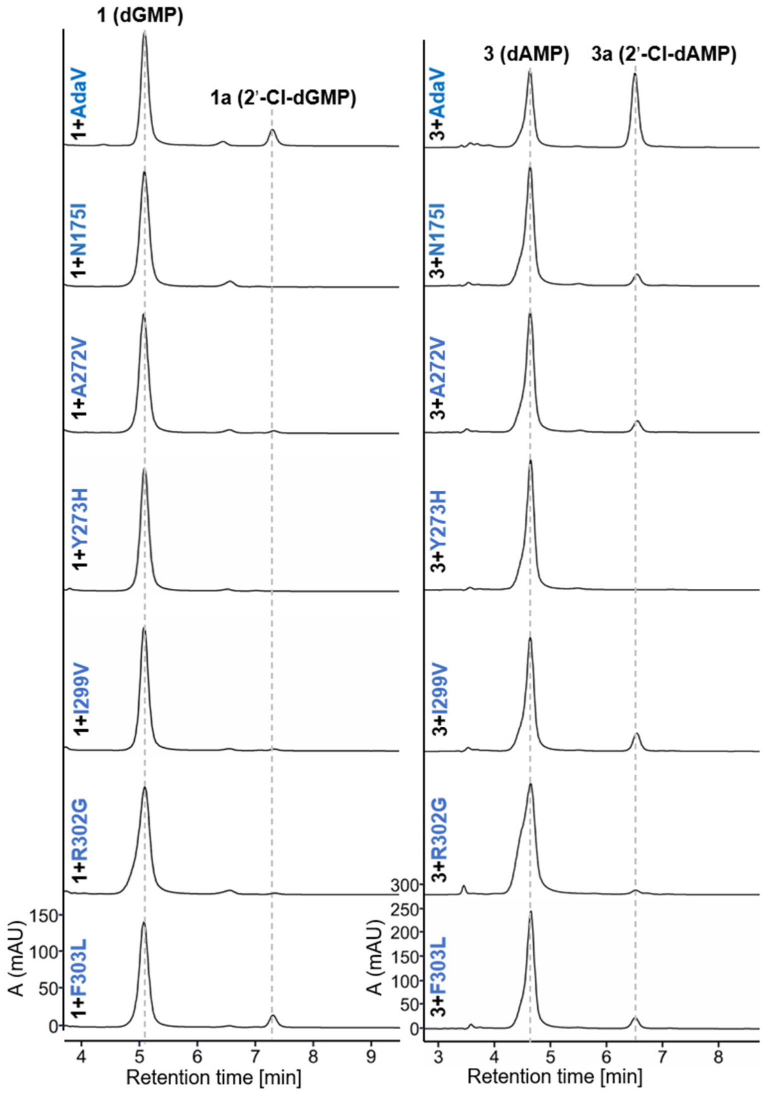

Supplementary Figure 37. HPLC analysis of enzymatic reactions catalyzed by N175I, A272V, Y273H, I299V, G302R and F303L variants of AdaV with dGMP (1) and dAMP (3). All HPLC spectra are representative of at least three experimental replicates.

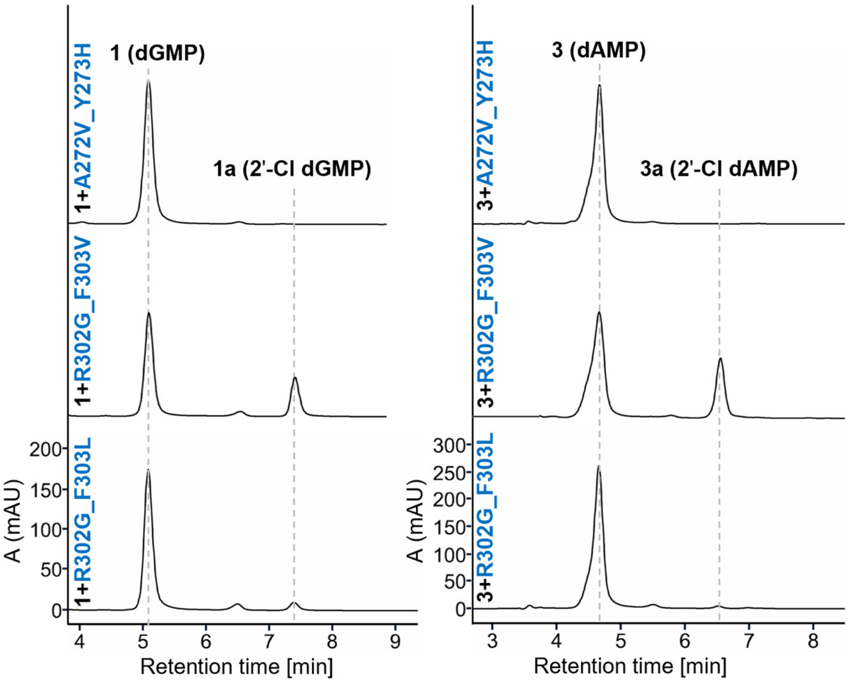

Supplementary Figure 38. HPLC analysis of enzymatic reactions catalyzed by A272V-Y273H, R302G-F303V and R302G-F303L variants of AdaV with dGMP (1) and dAMP (3). All HPLC spectra are representative of at least three experimental replicates.

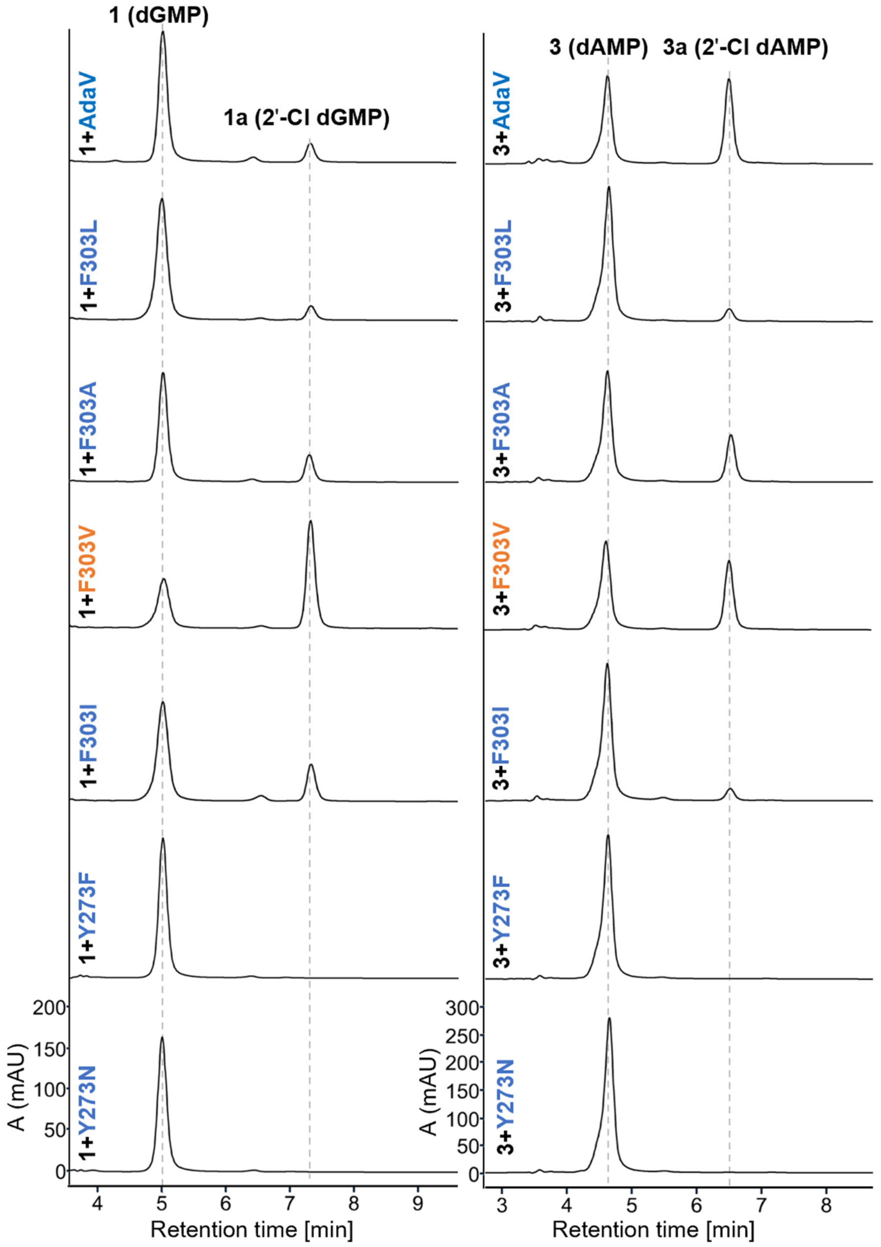

Supplementary Figure 39. HPLC analysis of enzymatic reactions catalyzed by F303L, F303A, F303V, F303I, Y273F and Y273N variants of AdaV with dGMP (1) and dAMP (3). All HPLC spectra are representative of at least three experimental replicates.

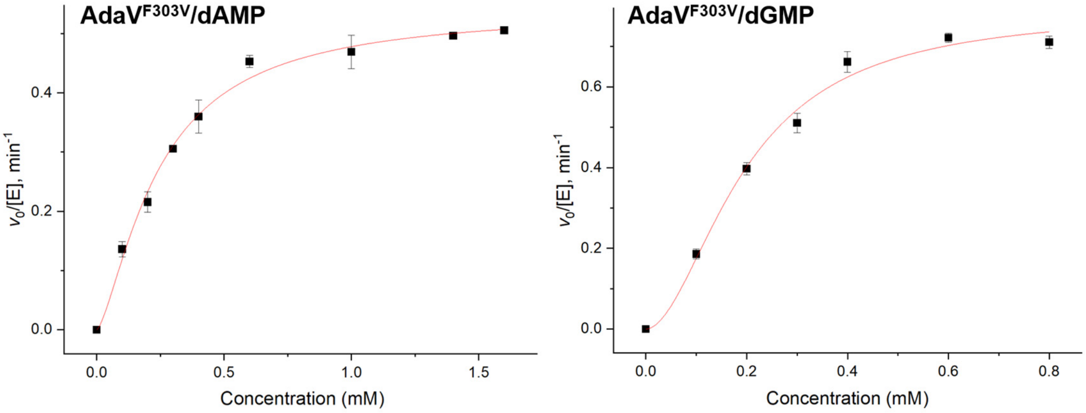

Kinetic Parameter	AdaV^{F303V}_dAMP	AdaV^{F303V}_dGMP
k_{cat} (min^{-1})	0.54 ± 0.02	0.79 ± 0.05
K_m (mM)	0.24 ± 0.02	0.2 ± 0.02
k_{cat}/K_m (mM^{-1}min^{-1})	2.30 ± 0.05	4.0 ± 0.1

Supplementary Figure 40. Steady-state kinetic analysis for AdaV^{F303V} with dGMP and dAMP as substrate. Table contains k_{cat}, K_m, and k_{cat}/K_m calculated by non-linear curve fitting to the Michaelis-Menten equation. Data are mean ± s.d. (n = 3 biologically independent experiments).

1.4 Role of the second-sphere residues – Figure S41 to S51

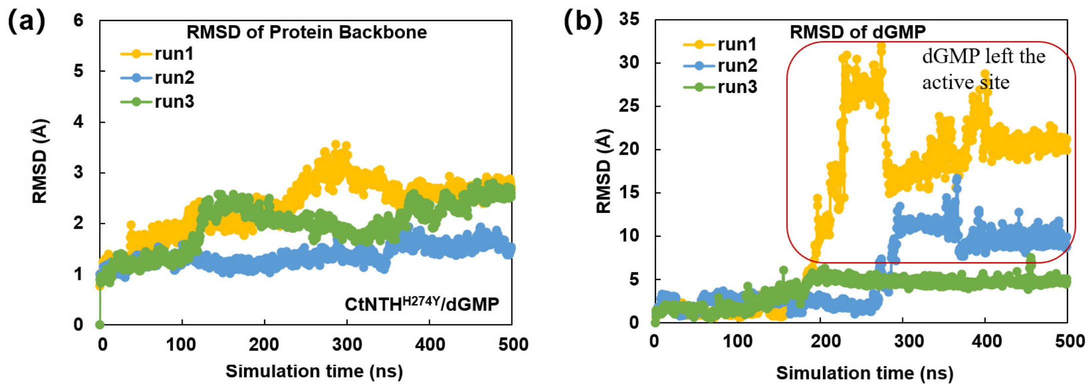

Supplementary Figure 41. RMSD curves of three replicas for MD of CtNTH^{H274Y}/dGMP. (a) RMSD of the protein backbone calculated relative to the first frame; (b) RMSD of dGMP calculated relative to the first frame.

In run 1 and run 2, dGMP moves away from the active site after 200 ns of simulation, suggesting that the binding of dGMP in the active site of CtNTH^{H274Y} is unstable.

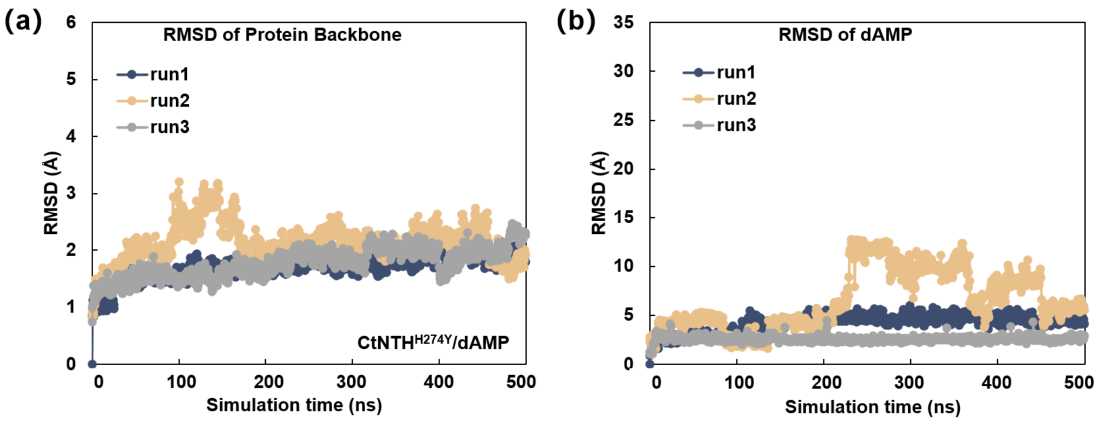

Supplementary Figure 42. RMSD curves of three replicas for MD of CtNTH^{H274Y}/dAMP. (a) RMSD of the protein backbone calculated relative to the first frame; (b) RMSD of dAMP calculated relative to the first frame.

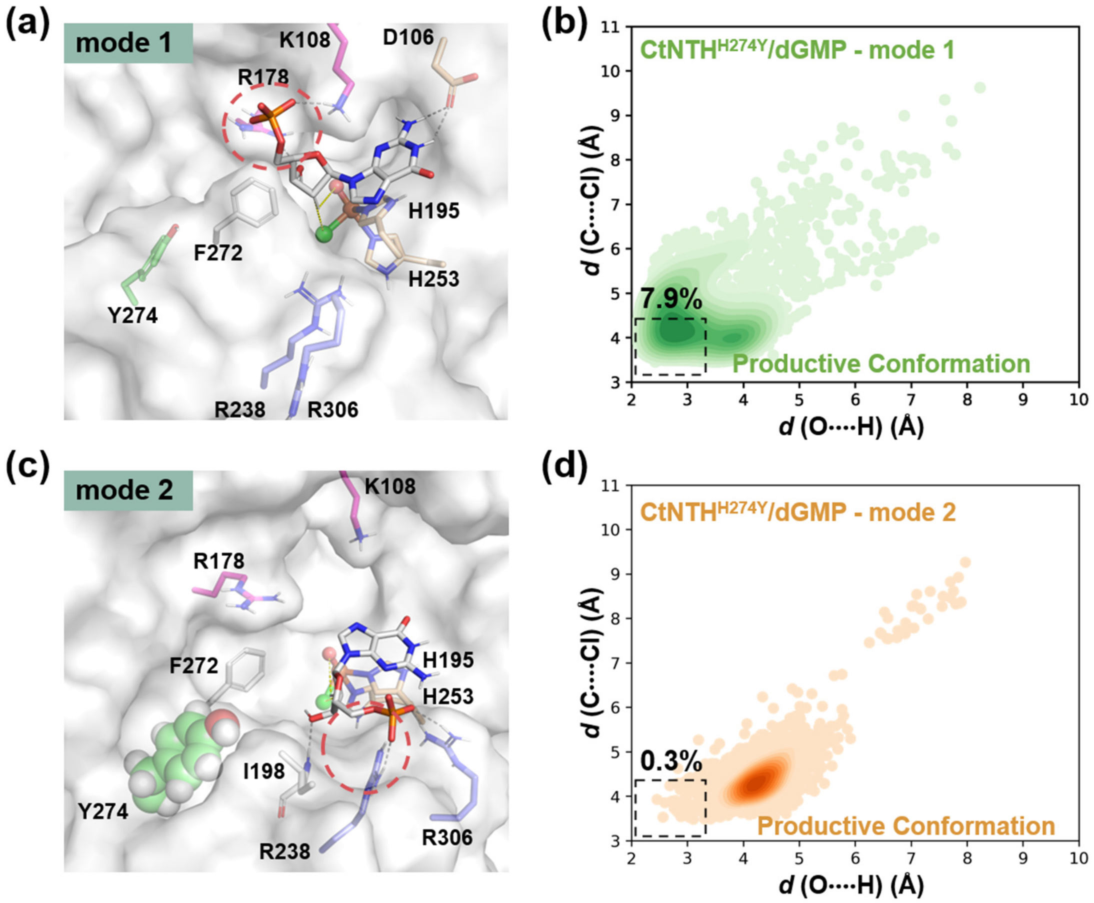

Supplementary Figure 43. CtNTH^{H274Y}/dGMP system: representative structures and statistical analysis of mode 1 and mode 2. Representative structures of mode 1 (a) and mode 2 (c). Distance distributions of C2′···Cl and O···H (in Å) in mode 1 (b) and mode 2 (d) of CtNTH^{H274Y}/dGMP. Two-dimensional plot of discrete points with Gaussian kernel density estimation of the distributions. The numbers in (b) and (d) donate the percentage of productive conformations of each mode in total frames. The productive conformations ($d_{\text{O-H}} \leq 3.2$ Å, $d_{\text{C-Cl}} \leq 4.5$ Å) are indicated with a dotted box.

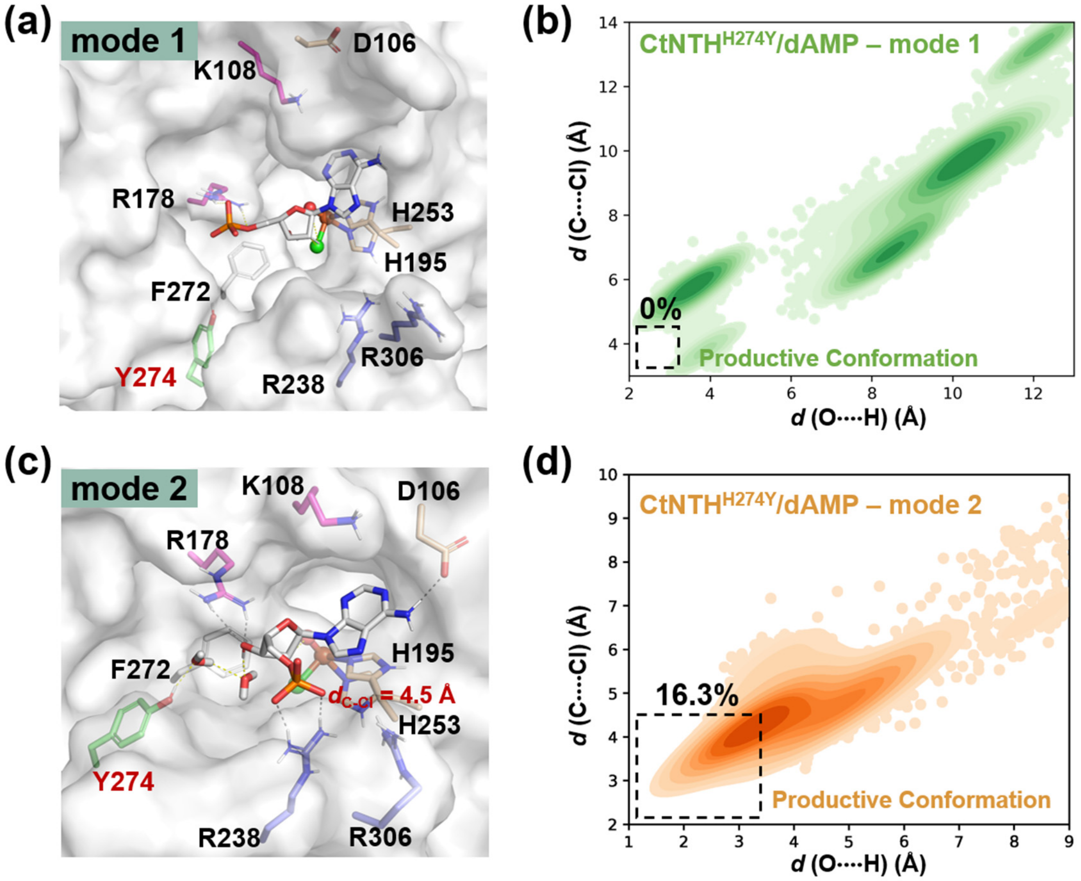

Supplementary Figure 44. CtNTH^{H274Y}/dAMP system: representative structures and statistical analysis of mode 1 and mode 2. Representative structures of mode 1 (a) and mode 2 (c). Distance distributions of C2′···Cl and O···H (in Å) in mode 1 (b) and mode 2 (d) of CtNTH^{H274Y}/dAMP. Two-dimensional plot of discrete points with Gaussian kernel density estimation of the distributions. The numbers in (b) and (d) donate the percentage of productive conformations of each mode in total frames. The productive conformations ($d_{O\text{-}H} \leq 3.2$ Å, $d_{C\text{-}Cl} \leq 4.5$ Å) are indicated with a dotted box.

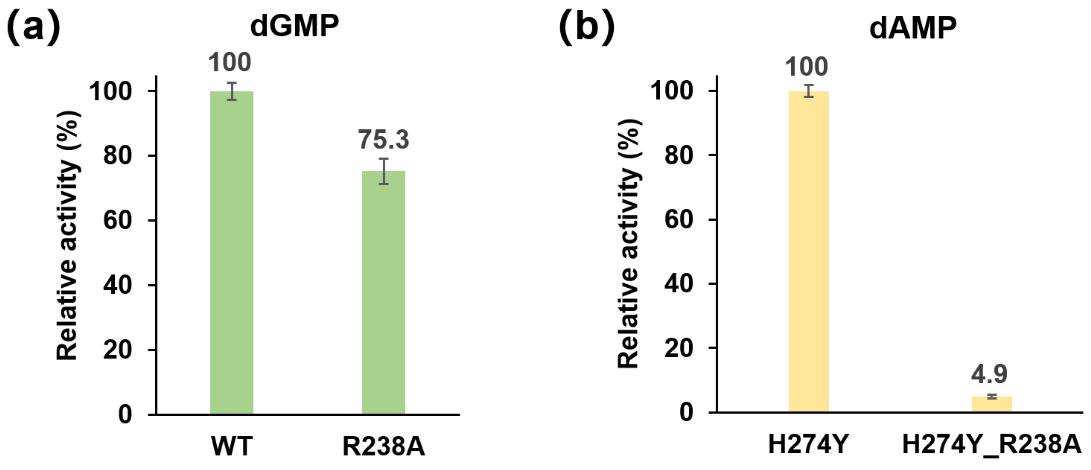

Supplementary Figure 45. (a) Relative halogenation activities of CtNTH^{WT} and CtNTH^{R238A} toward dGMP. (b) Relative halogenation activities of CtNTH^{H274Y} and CtNTH^{H274Y_R238A} toward dAMP.

The R238A mutation in the CtNTH^{WT}/dGMP and CtNTH^{H274Y}/dAMP systems has different effects on the chlorination activity. The former retained 75.3% chlorination activity, which indicates R238 is not indispensable to the binding of dGMP in CtNTH^{WT}. While the later only retained 4.9% chlorination activity, which suggests R238 is essential for the binding of dAMP in CtNTH^{H274Y} and thus supports binding mode 2.

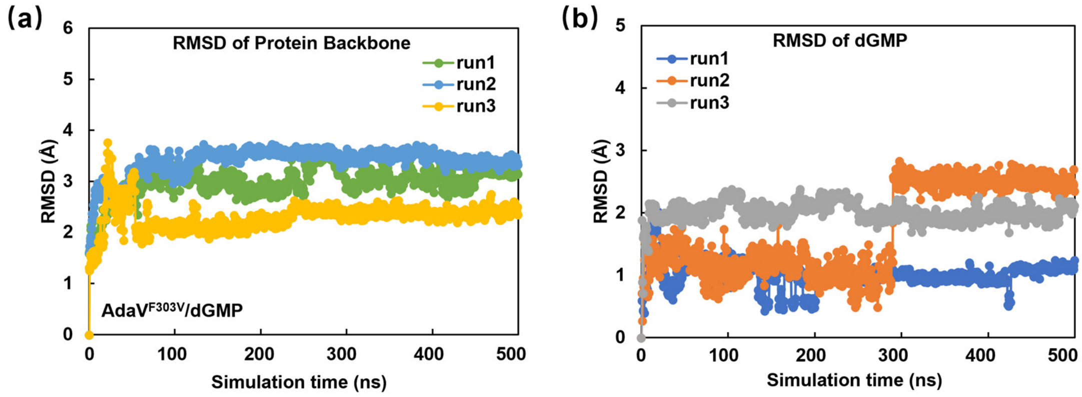

Supplementary Figure 46. RMSD curves of three replicas for MD of AdaV^{F303V}/dGMP. (a) RMSD of the protein backbone calculated relative to the first frame; (b) RMSD of dGMP calculated relative to the first frame.

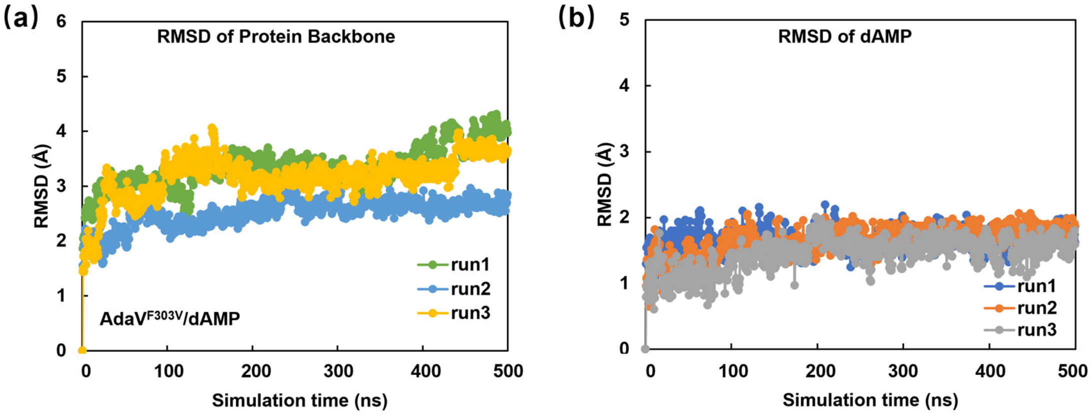

Supplementary Figure 47. RMSD curves of three replicas for MD of AdaV^{F303V}/dAMP. (a) RMSD of the protein backbone calculated relative to the first frame; (b) RMSD of dAMP calculated relative to the first frame.

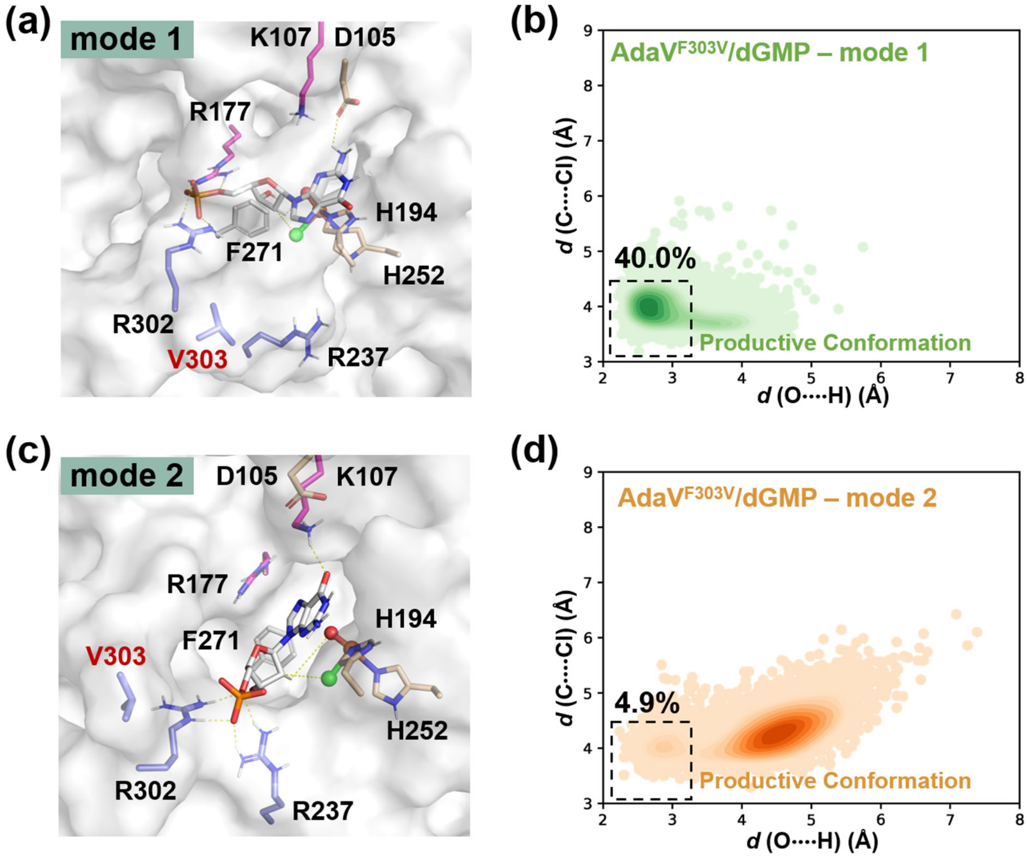

Supplementary Figure 48. AdaV^{F303V}/dGMP system: representative structures and statistical analysis of mode 1 and mode 2. Representative structures of mode 1 (a) and mode 2 (c). Distance distributions of C2′···Cl and O···H (in Å) in mode 1 (b) and mode 2 (d) of AdaV^{F303V}/dGMP. Two-dimensional plot of discrete points with Gaussian kernel density estimation of the distributions. The numbers in (b) and (d) donate the percentage of productive conformations of each mode in total frames. The productive conformations ($d_{O\text{-}H} \leq 3.2$ Å, $d_{C\text{-}Cl} \leq 4.5$ Å) are indicated with a dotted box.

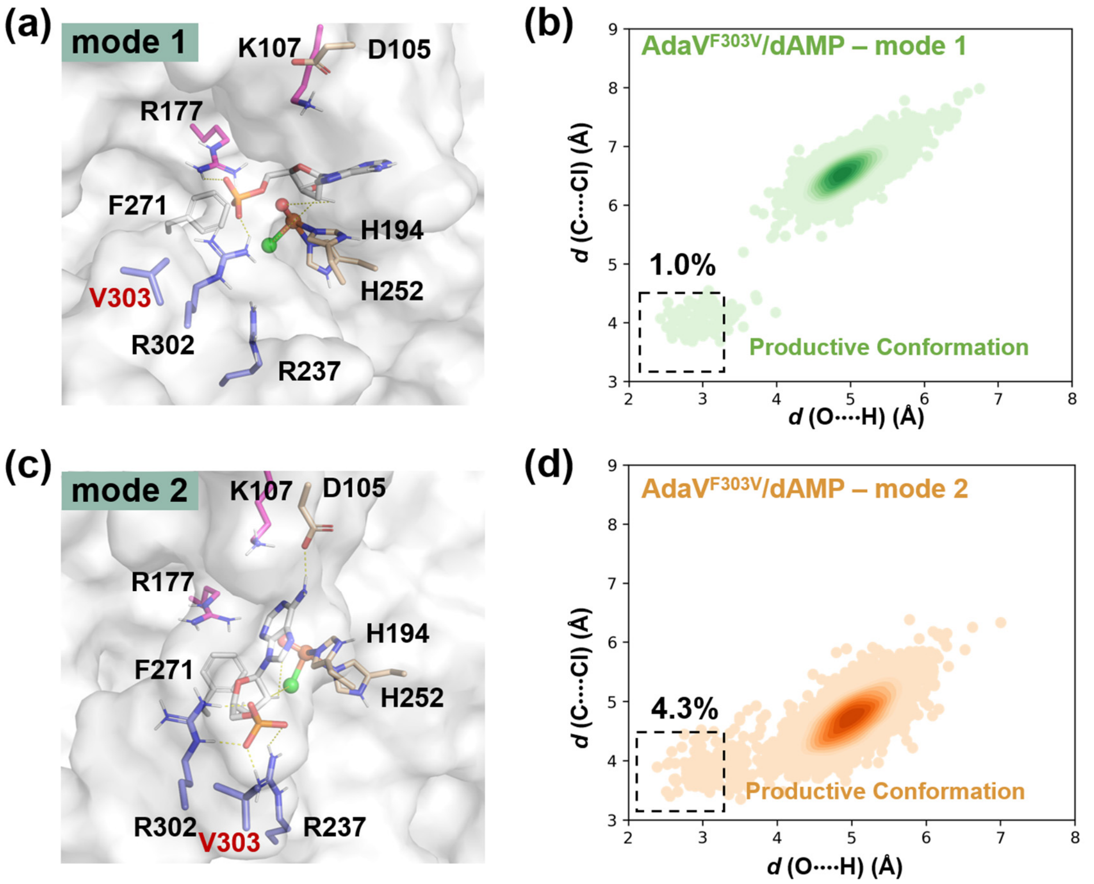

Supplementary Figure 49. AdaV^{F303V}/dAMP system: representative structures and statistical analysis of mode 1 and mode 2. Representative structures of mode 1 (a) and mode 2 (c). Distance distributions of C2′···Cl and O···H (in Å) in mode 1 (b) and mode 2 (d) of AdaV^{F303V}/dAMP. Two-dimensional plot of discrete points with Gaussian kernel density estimation of the distributions. The numbers in (b) and (d) donate the percentage of productive conformations of each mode in total frames. The productive conformations ($d_{O\text{-}H} \leq 3.2$ Å, $d_{C\text{-}Cl} \leq 4.5$ Å) are indicated with a dotted box.

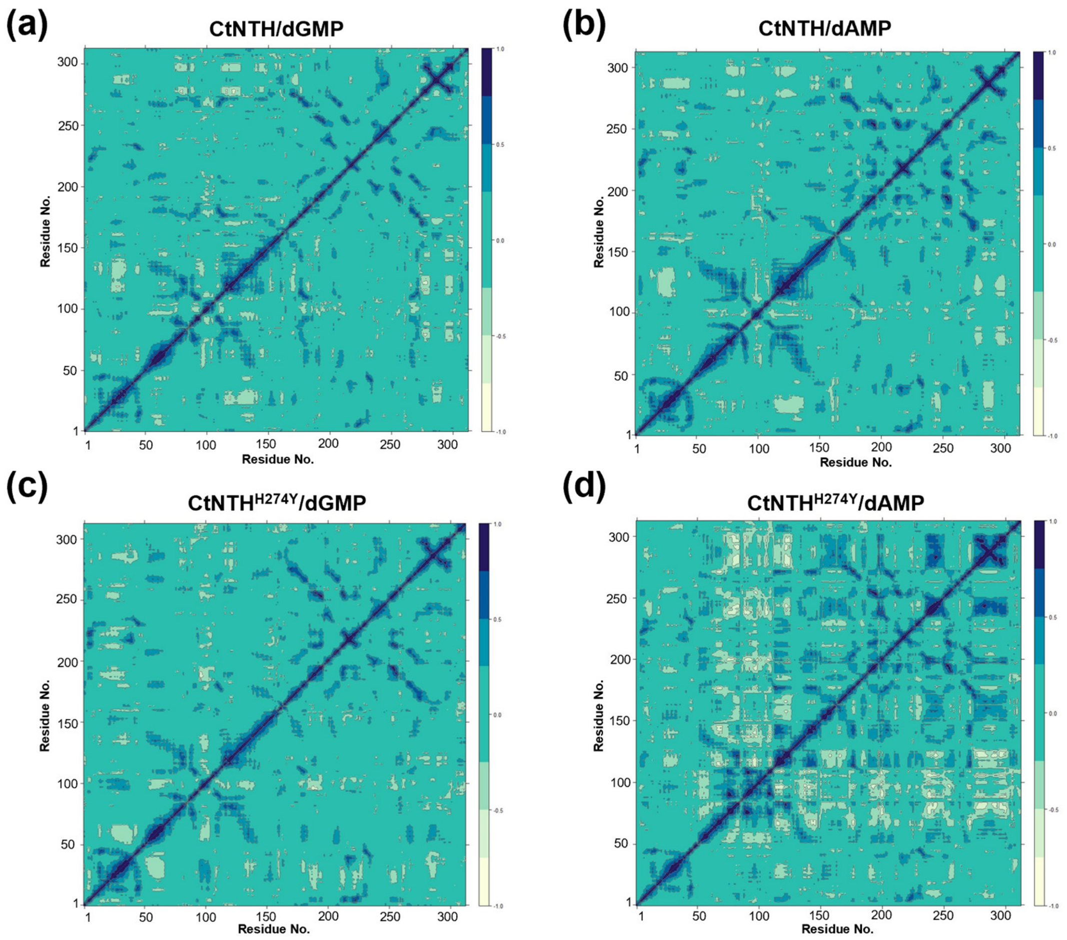

Supplementary Figure 50. Dynamic cross-correlation analysis of CtNTH and CtNTH^{H274Y}. (a) CtNTH with dGMP, (b) CtNTH with dAMP, (c) CtNTH^{H274Y} with dGMP and, (d) CtNTH^{H274Y} with dAMP. A positive correlation value (dark blue) indicates that the two protein parts move in the same direction. A negative value of correlation (cream) indicates that the direction of motion is in opposite directions.

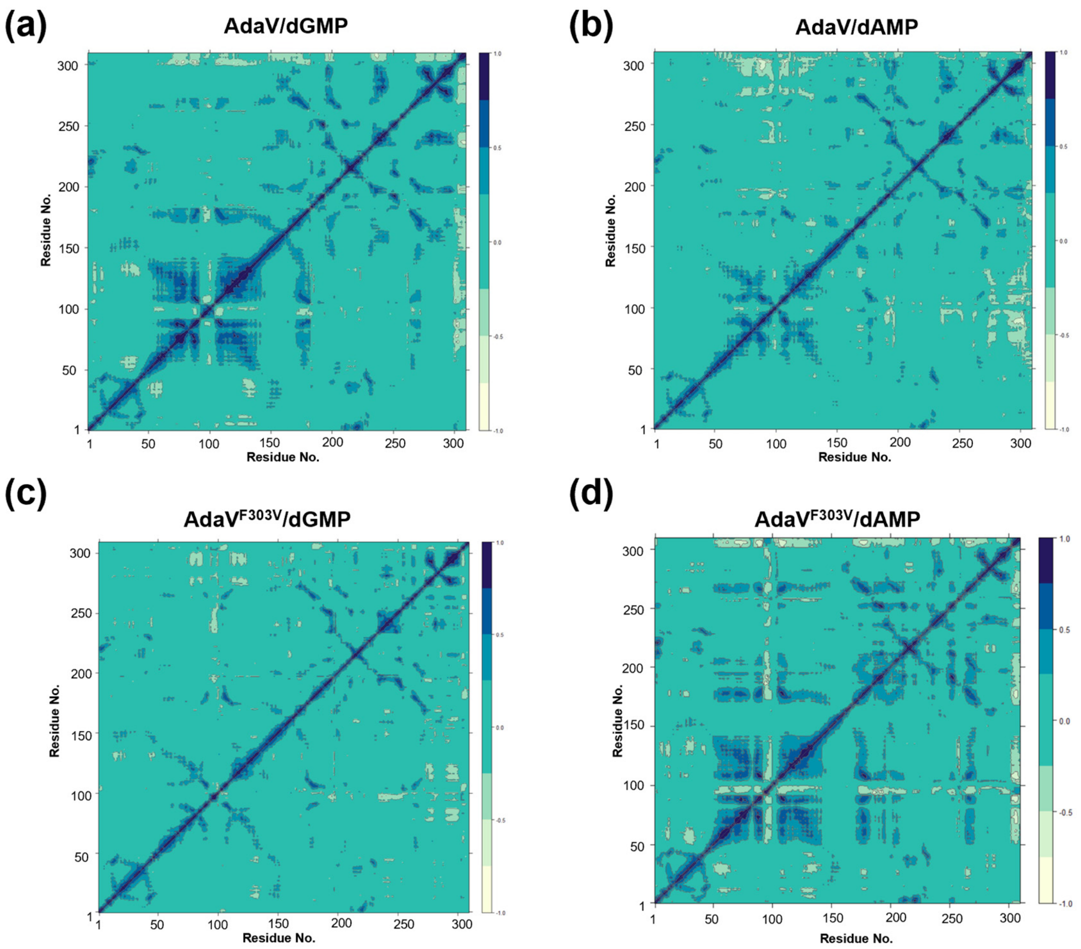

Supplementary Figure 51. Dynamic cross-correlation analysis of AdaV and AdaV^{F303V}. (a) AdaV with dGMP, (b) AdaV with dAMP, (c) AdaV^{F303V} with dGMP, and (d) AdaV^{F303V} with dAMP. A positive correlation value (dark blue) indicates that the two protein parts move in the same direction. A negative value of correlation (cream) indicates that the direction of motion is in opposite directions.

2. Supplementary Tables

Supplementary Table 1. Percent identity matrix of AdaV analogs. The sequence alignment was performed using EMBL-EBI[1]. 2, 5 and 8 were identified as dAMP halogenases in previous work[2].

	AdaV*	1	2*	3	4	5*	6	7	8*	9	10
AdaV *	100	86.7	85.5	86.3	86.8	73.8	75.2	70.0	67.0	55.5	51.0
MDF2704590.1 (1)	86.7	100	**88.2**	87.4	85.5	74.5	76.0	69.4	69.7	55.7	52.2
WP_182876399.1 (2) * (DI270)	85.5	88.2	100	89.5	84.7	75.5	75.6	70.6	65.9	54.5	50.7
WP_184541559.1 (3)	86.3	87.4	**89.5**	100	87.4	76.5	75.7	70.9	68.0	55.1	52.6
WP_225289932.1 (4)	**86.8**	85.5	84.7	87.4	100	72.0	74.2	69.9	65.0	53.2	51.9
WP_030883106.1 (5) * (ADK38)	73.8	74.5	75.5	76.5	72.0	100	72.6	72.7	69.8	54.2	51.4
WP_279554523.1 (6)	75.2	76.0	**75.6**	75.7	74.2	72.6	100	80.9	67.7	56.1	53.3
WP_189993169.1 (7)	70.0	69.4	70.6	70.9	69.9	**72.7**	80.9	100	66.4	54.9	51.0
WP_158075676.1 (8) * (BJP25)	67.0	69.7	65.9	68.0	65.0	69.8	67.7	66.4	100	54.7	53.7
WP_204007738.1 (9) (VaNTH)	**55.5**	55.7	54.5	55.1	53.2	54.2	56.1	54.9	54.7	100	67.3
WP_217394979.1 (10) (CtNTH)	51.0	52.2	50.7	52.6	51.9	51.4	53.3	51.0	53.7	67.3	100

Supplementary Table 2. Average distances of O···H and C2′···Cl in mode 1 and mode 2 for CtNTH/dGMP and AdaV/dAMP systems.

	**CtNTH/dGMP_mode 1**		**CtNTH/dGMP_mode 2**	
	average distance	standard deviation	average distance	standard deviation
O···H (Å)	2.8	0.4	3.1	0.5
C2'···Cl (Å)	4.2	0.3	5.1	0.7

	**AdaV/dAMP_mode 1**		**AdaV/dAMP_mode 2**	
	average distance	standard deviation	average distance	standard deviation
O···H (Å)	5.9	1.9	3.0	0.5
C2'···Cl (Å)	5.7	1.7	4.5	0.4

Supplementary Table 3. Statistical analysis of substrate binding modes and productive conformations. P1 represents the percentage of binding modes in total frames. P2 denotes the percentage of productive conformations of each mode in total frames.

CtNTH	dGMP		dAMP	
	P1	P2	P1	P2
mode 1	65.1%	43.5%	74.4%	2.1%
mode 2	8.9%	3.1%	20.4%	0.3%
other modes	26.0%	6.1%	5.2%	0.1%

AdaV	dGMP		dAMP	
	P1	P2	P1	P2
mode 1	28.2%	0%	24.5%	2.7%
mode 2	71.1%	2.9%	73.3%	25.2%
other modes	0.7%	0%	2.2%	2.3%

Other modes are primarily transitional binding mode that the phosphate group binds in the region between mode 1 and mode 2.

Supplementary Table 4. Statistical analysis of substrate binding modes and productive conformations in MD simulations of CtNTH^{H274Y} and AdaV^{F303V} with dGMP and dAMP. P1 represents the percentage of binding modes in total frames. P2 denotes the percentage of productive conformations of each mode in total frames.

CtNTH^{H274Y}	dGMP		dAMP	
	P1	P2	P1	P2
mode 1	20.8%	7.9%	32.5%	0%
mode 2	26.2%	0.3%	66.6%	16.3%
other modes	53.0%	0.4%	0.9%	0%

AdaV^{F303V}	dGMP		dAMP	
	P1	P2	P1	P2
mode 1	65.8%	40.0%	57.3%	1%
mode 2	32.8%	4.9%	33.3%	4.3%
other modes	1.4%	0%	9.4%	0.8%

Supplementary Table 5. Hydrogen bond occurrence between R302 and the phosphate group of the substrates in AdaV and AdaV^{F303V} systems.

	dGMP		dAMP	
	mode 1	mode 2	mode 1	mode 2
AdaV	99.4%	97.9%	86.8%	88.2%
AdaV^{F303V}	99.4%	99.7%	99.1%	93.6%

Supplementary Table 6. Correlation coefficient of residue 274 with F272 and substrates in CtNTH and CtNTH^{H274Y} systems. The correlation coefficient indicates the percentage of the correlated motions in the MD trajectories.

	F272	dAMP/dGMP (P atom)
CtNTH/dGMP	0.23	-0.71
CtNTH/dAMP	0.65	-0.80
CtNTH^{H274Y}/dGMP	0.61	-0.94
CtNTH^{H274Y}/dAMP	0.38	-0.55

Supplementary Table 7. Correlation coefficient of R302 with the phosphate group of dAMP/dGMP in AdaV and AdaV^{F303V} systems.

	F303/V303	dAMP/dGMP (P atom)
AdaV/dGMP	0.73	-0.73
AdaV/dAMP	0.84	-0.54
AdaV^{F303V}/dGMP	0.87	-0.45
AdaV^{F303V}/dAMP	0.58	-0.67

Supplementary Table S8. Harmonic restraints in MD simulations. Flat-bottom harmonic restraints of distance (in Å) values were applied in the first 100 ns MD simulations in all cases. The two restraints were the distance between reacting atoms, i.e., $d_{C2'\text{-}Cl}$ and $d_{O\text{-}H}$ (in Å). A moderate force constant of k=50 kcal/(mol·Å^2) was used.

	Distances (Å)			
	r1	r2	r3	r4
$d_{O\text{-}H}$	2.0	2.5	3.5	4.0
$d_{C2'\text{-}Cl}$	2.5	3.0	4.5	5.0

3. Supplementary Notes

3.1 Primers used in this study

Primers	Sequence (5'—3')
R99A-F	AGC GAA GTG CCG GCA CTG GCG
R99A-R	GCC GTT CGC CAG TGC CGG CA C
D106A-F	AAC GGC GTG GCG GCA CCG AAA
D106A-R	AAA TTC TTT CGG TGC CGC CAC
K108A-F	GTG GCG GAT CCG GCA GAA TTT
K108A-R	ATG CAG AAA TTC TGC CGG AT C
R178A-F	AGC AGC ATT CTG GCC GTG ATT
R178A-R	ATA ATG AAT CAC GGC CAG AAT
I198A-F	GAA CAT AGC GGC GCA CAG ATG
I198A-R	GCC CAG CAT CTG TGC GCC GCT
Q199A-F	CAT AGC GGC ATT GCA ATG CTG
Q199A-R	CAC GCC CAG CAT TGC AAT GCC
F272A-F	AGC AGT GTT CTG GCA GCA TAT
F272A-R	CTG CGG ATA TGC TGC CAG AAC
H193A-F	GAA ATC CTG GCG GCA GAA CAT
H193A-R	GCC GCT ATG TTC TGC CGC CAG
H195A-F	CTG GCG CAT GAA GCA AGC GGC
H195A-R	CTG AAT GCC GCT TGC TTC ATG
H253A-F	CGC CCG AGT ACC GCA CGC GTG
H253A-R	GCG ATG CAC GCG TGC GGT ACT
Q204A-F	ATG CTG GGC GTG GCA TTT CCG
Q204A-R	GCT CGC CGG A AA TGC CAC GCC
R306A-F	AAC AAA GGC CTG GCA GAG CTG
R306A-R	CAG GCC CAG CTC TGC CAG GCC
I176N-F	CGC CAT AGC AGC AAT CTG CGC
I176N-R	AAT CAC GCG CAG ATT GCT GCT
V273A-F	AGC GTG GTG TTT GCA CAT CCG
V273A-R	ATG ATC CGG ATG TGC AAA CAC
H274Y-F	GTG GTG TTT GTG TAT CCG GAT
H274Y-R	GCT ATG ATC CGG ATA CAC AAA
V301I-F	TGG GGC GCG TTT ATC AAC AAA
V301I-R	CAG GCC TTT GTT GAT AAA CGC
L304F-F	C AAA GGC TTT CGC GAG CTG GGC CTG AGT
L304F-R	TC GCG AAA GCC TTT GTT CAC AAA CGC GC
G304R-F	G AAC AAA CGT CTG CGC GAG CTG GGC CTG
G304R-R	CG CAG ACG TTT GTT CAC AAA CGC GCC CC
V273A_H274Y-F	AGC GTG GTG TTT GCA TAT CCG GAT
V273A_H274Y-R	GCT ATG ATC CGG ATA TGC AAA CAC
H274A-F	GTG GTG TTT GTG GCA CCG GAT
H274A-R	GCT ATG ATC CGG TGC CAC AAA
H274F-F	GTG GTG TTT GTG TTC CCG GAT
H274F-R	GCT ATG ATC CGG GAA CAC AAA
AdaV-N175I-F	CAT GAT GCC ACC ATT CTG CGC
AdaV-N175I-R	AAT AAC GCG CAG AAT GGT GGC
AdaV-A272V-F	AGT GTT CTG TTT GTG TAT CCG
AdaV-A272V-R	ATG CTG CGG ATA CAC AAA CAG
AdaV-Y273H-F	GTT CTG TTT GCA CAT CCG CAG

AdaV-Y273H-R	TTT ATG CTG CGG ATG TGC AAA
AdaV-I299V-F	TGG GGT GAT TTT GTG GAT AGT
AdaV-I299V-R	AAA GCG ACT ATC CAC AAA ATC
AdaV-R302G-F	T GAT AGT GGC TTT CAG GGC CTG GGT AAA C
AdaV-R302G-R	TG AAA GCC ACT ATC AAT AAA ATC ACC CCA GG
AdaV-F303L-F	T AGT CGC TTA CAG GGC CTG GGT AAA CAG AG
AdaV-F303L-R	CC CTG TAA GCG ACT ATC AAT AAA ATC ACC CCA G
AdaV-F303A-F	T AGT CGC GCA CAG GGC CTG GGT AAA CAG AG
AdaV-F303A-R	CC CTG TGC GCG ACT ATC AAT AAA ATC ACC CCA G
AdaV-F303V-F	T AGT CGC GTT CAG GGC CTG GGT AAA CAG AG
AdaV-F303V-R	CC CTG AAC GCG ACT ATC AAT AAA ATC ACC CCA G
AdaV-F303I-F	T AGT CGC ATT CAG GGC CTG GGT AAA CAG AG
AdaV-F303I-R	CC CTG AAT GCG ACT ATC AAT AAA ATC ACC CCA G
AdaV-Y273F-F	GTT CTG TTT GCA TTT CCG CAG
AdaV-Y273F-R	TTT ATG CTG CGG AAA TGC AAA
AdaV-Y273N-F	GTT CTG TTT GCA AAT CCG CAG
AdaV-Y273N-R	TTT ATG CTG CGG ATT TGC AAA
AdaV_A272V_Y273H-F	AGT GTT CTG TTT GTG CAT CCG CAG
AdaV_A272V_Y273H-R	TTT ATG CTG CGG ATG CAC AAA CAG
AdaV_R302G_F303L_F	AT AGT GGC TTA CAG GGC CTG GGT AAA CAG
AdaV_R302G_F303L_R	CCTG TAA GCC ACT ATC AAT AAA ATC ACC CCA GG
AdaV_R302G_F303V_F	AT AGT GGC GTT CAG GGC CTG GGT AAA CAG
AdaV_R302G_F303V_R	C CTG AAC GCC ACT ATC AAT AAA ATC ACC CCA GG

3.2 DNA and amino acid sequences

The DNA sequence of VaNTH:
ATGACCGATGTGGTGCCGATGCGCCTGCAAGGCGAAGTGCCGGAACTGCG
CATGGAAAGCATTGAAGAAGATGCGCATCGCAACCTGGGCCGCTTTCGCG
AACAGCTGGATACCTGGGGCTTTATGGCGCTGGAAATGCCGAGCATTGGCG
CGCGCGTGGATCGCCTGTATAAAGCGTTTGATGCGGCGCTGAAAAGCACGA
GCCCGAGCCTGGCGGAATTTGCGACCGCGAAAACCCCGCAAGCGACCCCG
GGCGGCAACCATGGCTTTTTTCCGTTTGAAAGCGAAGTGCCGCGCCTGGC
GGCGGGCGTGCCGGATCCGAAAGAATTTATGCATGTTAGCGGCGCGATGCT
GGATGATGTGCCGCCGGGCGCGGCCGCGATGCTGACCGCGTTTCCGGATCT
GGCGGATCATAGCCGCTTTCTGTTTGAAACCGCGTTTCGCGTGGCGCAAGC
GCTGGGCGGCGTGGTGCTGGAACTGCTGCCGGGCGAACCGCCGAAACTGG
ATCTGAGCGCGTATAGCAGCATTCTGCGCGTGATTCATTATCGCGATCCGGA
TCGCCGCGAAGTGCTGGCGCATGAACATAGCGGCATTCAGATGGTGGGCGT
GCAGCTGCCGCCGAGCGAAGGCGGCCTGCAGTATGTGCTGAACGATGGCA
CCTGGGTGGAACCGGTGATTCAAGGCACCGATGTGCTGCTGTTTAATATTG
GTCGCATGCTGAGCACCGCGAGCGGCGGCCGCGTGCGCCCAAGTACCCAC
CGTGTGCATCGTAGCCCGCTGGCGACGAGCGTGGAACGCTGGAGCAGCGT
GCTGTTTGTGCATCCGAACCATGAAGATCCGCAGTGGTGGATGGATGGCGA
TGGCAACACCGTGGTGAGCGATGCGACCTGGGGCGATTTTGTGCATAAAG
GCCTGAACGAACTGGGCCTGACCGATTAA
The amino acid sequence of VaNTH:
MTDVVPMRLQGEVPELRMESIEEDAHRNLGRFREQLDTWGFMALEMPSIGA

RVDRLYKAFDAALKSTSPSLAEFATAKTPQATPGGNHGFFPFESEVPRLAAGVP
DPKEFMHVSGAMLDDVPPGAAAMLTAFPDLADHSRFLFETAFRVAQALGGV
VLELLPGEPPKLDLSAYSSILRVIHYRDPDRREVLAHEHSGIQMVGVQLPPSEG
GLQYVLNDGTWVEPVIQGTDVLLFNIGRMLSTASGGRVRPSTHRVHRSPLATS
VERWSSVLFVHPNHEDPQWWMDGDGNTVVSDATWGDFVHKGLNELGLTD

The DNA sequence of CtNTH:

ATGAGCGAAGTGGAACTGCTGCCGCTGGCGGGCAAAGTGCCGCAGCTGCA
TGTGGAAAGCGTGGAAGATGATGCGATTCGCAACATTGAAGAATTTCGCGA
ACAGCTGGCGACCTGGGGCTTTATGGCGCTGGAAGTGCCGGGCATTGGCG
ATCGCGTGACCGAACTGTATGGCGCGTTTGATGCGGCGCTGGCGAGCACGA
GCCCGAGCCTGTATGAATTTGCGGTGGATCGCGTGCCGCAAGCGAGCGCG
GGCGGCAACCATGGCTTTTTTCGCCCGGGCAGCGAAGTGCCGCGCCTGGC
GAACGGCGTGGCGGATCCGAAAGAATTTCTGCATGTGAGCGGCGCGATGC
TGGATAACCATCCGGCGGGCAGCGCGGCGCTGCTGGAAGCGTTTCCGGCG
CTGGCGGAACAGAGCCGCTTTATTTTTGAACTGGGCATTCGCGTGGCGGCG
AGCCTGGGCGATGTGGTGCGCGAGATTCTGCCGGGCCAAGCGCCGGAACT
GGGCCTGAGCCGCCATAGCAGCATTCTGCGCGTGATTCATTATCAAGATAGT
CAGCGCCGCGAAATCCTGGCGCATGAACATAGCGGCATTCAGATGCTGGGC
GTGCAGTTTCCGGCGAGCGAGGGTGGCCTGCAGTATATTCTGAACGATGGC
ACCTGGGTGGAACCGATTATTTGGGGCACCGATGTGCTGCTGTGCAACATT
GGTCGCATGTTAAGCGAGGCGAGTGGCCGTCGCGTTCGCCCGAGTACCCAT
CGCGTGCATCGCAGCCCGATGGCGATGAGCGAACGCCGCTGGAGCAGCGT
GGTGTTTGTGCATCCGGATCATAGCGGCGAACAGTGGACCATTACCGAAGA
GGAACAAGTGCGCATGCTGGGCGAACCGTGGGGCGCGTTTGTGAACAAAG
GCCTGCGCGAGCTGGGCCTGAGTGATTAA

The amino acid sequence of CtNTH:

MSEVELLPLAGKVPQLHVESVEDDAIRNIEEFREQLATWGFMALEVPGIGDRV
TELYGAFDAALASTSPSLYEFAVDRVPQASAGGNHGFFRPGSEVPRLANGVAD
PKEFLHVSGAMLDNHPAGSAALLEAFPALAEQSRFIFELGIRVAASLGDVVREI
LPGQAPELGLSRHSSILRVIHYQDSQRREILAHEHSGIQMLGVQFPASEGGLQY
ILNDGTWVEPIIWGTDVLLCNIGRMLSEASGRRVRPSTHRVHRSPMAMSERR
WSSVVFVHPDHSGEQWTITEEEQVRMLGEPWGAFVNKGLRELGLSD

3.3 ^{1}H NMR and ^{13}C NMR of 2′-Cl-dGMP

^{1}H NMR (500 MHz, DMSO-*d6*) δ 7.95 (s, 1H), 6.60 (s, 1H), 5.98-5.96 (d, J = 7.3 Hz, 1H), 5.00 – 4.97 (m, 1H), 4.34 (s, 1H), 4.16 (s, 1H), 4.08 (m, 1H), 4.00 (m, 1H).
^{13}C NMR (126 MHz, DMSO-*d6*) δ 157.09, 154.37, 151.84, 135.59, 116.92, 86.82, 84.10, 70.56, 65.41, 61.17.

4. Supplementary References

1. Madeira, F. *et al.* Search and sequence analysis tools services from EMBL-EBI in 2022. *Nucleic Acids Res.* **50**, W276-W279 (2022).

2. Zhai, G. *et al.* Structural insight into the catalytic mechanism of non-heme iron halogenase AdaV in 2′-chloropentostatin biosynthesis. *ACS Catal.* **12**, 13910-13920 (2022).
